# Synergistic Dual-Cure Reactions for the Fabrication of Thermosets by Chemical Heating

**DOI:** 10.1021/acssuschemeng.4c01965

**Published:** 2024-08-02

**Authors:** Michael L. McGraw, Bennett Addison, Ryan W. Clarke, Robert D. Allen, Nicholas A. Rorrer

**Affiliations:** Renewable Resources and Enabling Sciences Center, National Renewable Energy Laboratory, Golden, Colorado 80401, United States

**Keywords:** energy efficiency, manufacturing, composite synthesis, thermosets, dual cure, chemical heating, recyclable-by-design

## Abstract

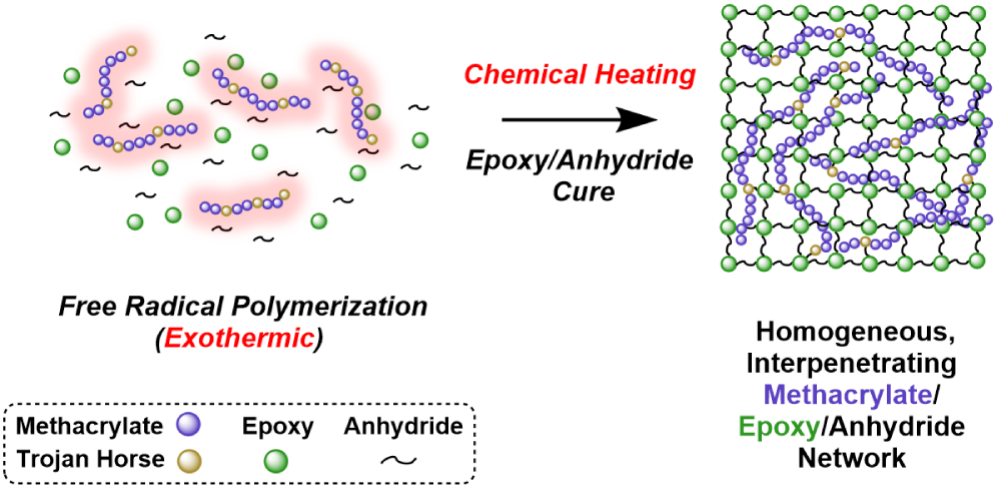

Large composite structures, such as those used in wind energy applications, rely on the bulk polymerization of thermosets on an impressively large scale. To accomplish this, traditional thermoset polymerizations require both elevated temperatures (>100 °C) and extended cure durations (>5 h) for complete conversion, necessitating the use of oversize ovens or heated molds. In turn, these requirements lead to energy-intensive polymerizations, incurring high manufacturing costs and process emissions. In this study, we develop thermoset polymerizations that can be initiated at room temperature through a transformative “chemical heating” concept, in which the exothermic energy of a secondary reaction is used to facilitate the heating of a primary thermoset polymerization. By leveraging a redox-initiated methacrylate free radical polymerization as a source of exothermic chemical energy, we can achieve peak reaction temperatures >140 °C to initiate the polymerization of epoxy–anhydride thermosets without external heating. Furthermore, by employing Trojan horse methacrylate monomers to induce mixing between methacrylate and epoxy–anhydride domains, we achieve the synthesis of homogeneous hybrid polymeric materials with competitive thermomechanical properties and tunability. Herein, we establish a proof-of-concept for our innovative chemical heating method and advocate for its industrial integration for more energy-efficient and streamlined manufacturing of wind blades and large composite parts more broadly.

## Abstract 2

This work introduces the concept of chemical heating, which utilizes an exothermic polymerization to cure an epoxy−anhydride thermoset.

## Introduction

In an effort to protect the environment and steward nature’s precious resources, the polymer community has been concerned with the energy and carbon demands of our products. Great advancements have been made in the sourcing^1−9^ of polymers from renewable feedstocks (*e.g.*, biomass, waste plastics, municipal solid waste, *etc.*) designed for a circular economy.^10−19^ Despite the tremendous progress made in the sourcing of polymers, there is minimal consideration of the manufacturing demands of the polymerization process, which accounts for significant fractions of the total energy cost and the CO_2_ emissions of polymer products.^20−30^ In particular, thermosets (cross-linked polymers) incur a relatively high energy and emissions cost associated with their production. For example, epoxy-amine resins require ∼75 MJ/kg in sourcing the monomer feedstock, while requiring 60–70 MJ/kg in process fuel and electricity across their production.^27^ Because of this reality, we set out to develop innovative technologies to both save energy and reduce emissions from a processing and manufacturing standpoint.

Most of the energy cost associated with process fuel and electricity is related to heating.^20,27,28,30^ However, thermosetting reactions are generally exothermic and produce heat as the reaction proceeds. Therefore, it is possible to envision scenarios where reactions simply produce their own heat as they proceed and can heat themselves in a self-sustaining way. Unfortunately, this is generally not the case, because monomer reactivities at room temperature (RT) are too low to produce meaningful heating. Additionally, as thermoset polymerizations proceed, chains become entangled/networked, collision frequency decays, and overall reactivity slows exponentially through a phenomenon known as vitrification.^31−33^ Vitrification further demands higher temperatures to be employed over the course of a reaction to ensure that the operating temperature for cure is always above the ever-increasing glass transition temperature (*T*_g_). For these reasons, reliance on the natural exotherm is generally unrealistic, and supplementary energy input is almost always required.

To overcome these issues for thermosetting reactions, we envisioned a more ideal dual cure (DC) scenario wherein a second auxiliary polymerization reaction—one that is highly exothermic and spontaneous at RT—is introduced in parallel to the thermoset reaction to provide *chemical heating* to the thermoset monomers. This chemical heating should not only reduce the necessity for external heating but also heat the reaction evenly throughout as opposed to ovens or conventional heating element which lead to significant heat gradients from the surface of the material to its depths. For this second auxiliary reaction, we hypothesized that (meth)acrylate-free radical polymerization would be an appropriate choice for several reasons. First and foremost, the exothermic nature of (meth)acrylate polymerizations^34−36^ and the ease of initiating them at room temperature^37^ is key to facilitating reliable chemical heating, while the nature of their free-radical polymerization is known to be tolerant to many functional groups such as alcohols, amines, carboxylic acids, water, and more.^38^ Additionally, (meth)acrylate monomers are inexpensive, commercially available, and highly diverse in structure/functionality, which not only ensures their practicality but also enables control over the intensity of chemical heating, the mechanical performance, and the microstructure of the final material.

The candidate thermoset system we chose for this study is epoxy/anhydride, which has gained recent popularity due to its sustainability evaluation compared to more conventional epoxy systems.^39−61^ As an example, Wang *et al.* recently published on a bioderivable epoxy/anhydride resin, named *P*oly-*E*ster *C*ovalent *A*daptable *N*etwork (PECAN), which was designed in analogy to a conventional epoxy/amine formulation used for wind blades which demonstrated up to 40% lower GHG emissions and recyclability while maintaining all requisite performance metrics.^62^ Despite the benefits of this system, sluggish initiation at temperatures below 80 °C coupled with long cure times (>5 h) may be prohibitive to its widespread adoption by industry. Therefore, successful chemical heating and subsequent curing of PECAN without the aid of external heating elements would both represent the necessary proof of concept for the chemical heating idea and a meaningful advancement of a promising new thermoset technology.

**Figure 1:**
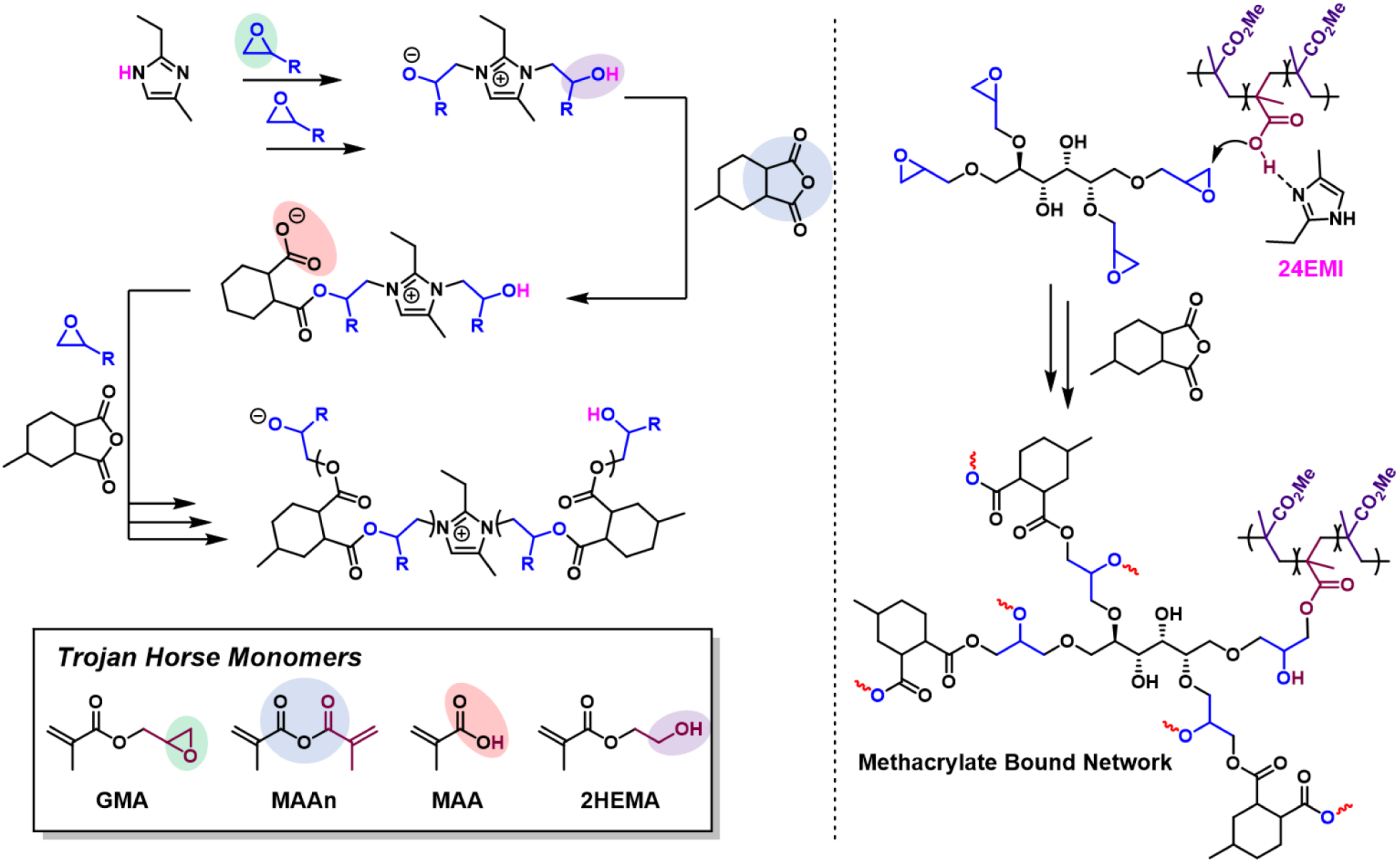
Chemical representations of dualcure chemistry. (Left) A general schematic of the PECAN curing mechanism.^63−70^ Emphasized is that the TH monomers mimic the functional groups involved in the PECAN polymerization mechanism. (Right) A proposed mechanism by which a TH (MAA) containing methacrylate chain binds into a PECAN network and participates in cross-linking. Abbreviations: 24EMI, 2-ethyl-4-methylimidazole; GMA, glycidyl methacrylate; MAAn, methacrylic anhydride; MAA, methacrylic acid; 2HEMA, 2-hydroxyethyl methacrylate.

By combining both methacrylate and PECAN monomers into one system, one would expect the methacrylate polymer chains to separate into their own domains. Thus, we designed a Trojan horse (TH) system to introduce covalent bonding of the methacrylate chains into the PECAN network to force mixing and homogeneity between the methacrylate/PECAN domains. This method involves incorporation of a small percentage of functionalized TH methacrylate monomer among a majority of nonfunctional methyl methacrylate (MMA) or ethyl methacrylate (EMA). The general logic is to employ functionalized TH monomers bearing functional groups that are operative in the PECAN propagation cycle. For example, epoxides, anhydrides, carboxylic acids, and alcohols are all active in the PECAN propagation cycle. Methacrylic acid (MAA), if present during PECAN polymerization could plausibly be in equilibrium with the carboxylates of the growing PECAN network and thus incorporate itself into the network by attacking and adding to an epoxide (Figure 1). Four TH monomers were investigated in this work: MAA, methacrylic anhydride (MAAn), glycidyl methacrylate (GMA), and 2-hydroxyethyl methacrylate (2HEMA). While they all work comparatively well in homogenizing the PECAN/methacrylate network, our preference for the carboxylic acid-containing TH is founded on the fact that MAA is extremely cheap, ubiquitous, and relatively safe to work with and was thus the primary focus of this investigation. The alcohol-dependent monomer 2HEMA seems to work equally well. MAAn has sluggish polymerization kinetics and does not allow the methacrylate to polymerize to full conversion. GMA is a known carcinogen and thus was avoided.

While we acknowledge that several other groups have previously disclosed similar dual-cure technologies,^71−87^ to our knowledge, none of these technologies leverage the thermodynamics of polymerization reactions to produce energy efficiency advantages. Like DC, the broad field of frontal polymerization^28,88−98^ (FP) leverages the enthalpy of polymerization to *specifically initiate polymerization*. In FP, a stimulus (such as heat) is applied at some point or plane to initiate polymerization. Then, the polymerization exotherm conducts outward from the starting point and continues activating the initiator as the polymerization “front” moves through the 3-dimensional bulk. One could argue that this is a form of chemical heating since the polymerization exotherm is used to activate the initiator. The DC technology presented here is differentiated from FP because the chemical heating generated in DC is used to support the initiation and propagation of the thermoset polymerization and to keep the growing polymers above their *T*_g_ to avoid vitrification limitations.

Thus, by combining a thermoset resin with a (meth)acrylate-based free-radical polymerization, we created a DC system. One in which the radical chain growth reaction can be initiated through redox, moderate thermal, or UV light to then produce thermal energy for a second reaction to initiate/propagate through thermal means and continue due to its own exotherm. Perhaps most intriguingly, we designed a simple chemical handle to provide control over the morphological consequences of this two-polymer system. Finally, we have provided proof of concept demonstrating that ∼25 wt % methacrylate incorporation in our candidate PECAN formulation is sufficient to heat and completely cure the material without the necessity for external heating and in a timely manner. We believe that what we have learned through this investigation suggests a paradigm shift in thermoset synthesis and that this technology can be easily translated to many other thermoset systems beyond PECAN. Herein, we disclose the results of our investigation into a synergistic DC system as well as the characterization of a diverse class of materials that demonstrate high-performance and tunability.

## Results

### Background, Design, and Synthesis

**Figure 2:**
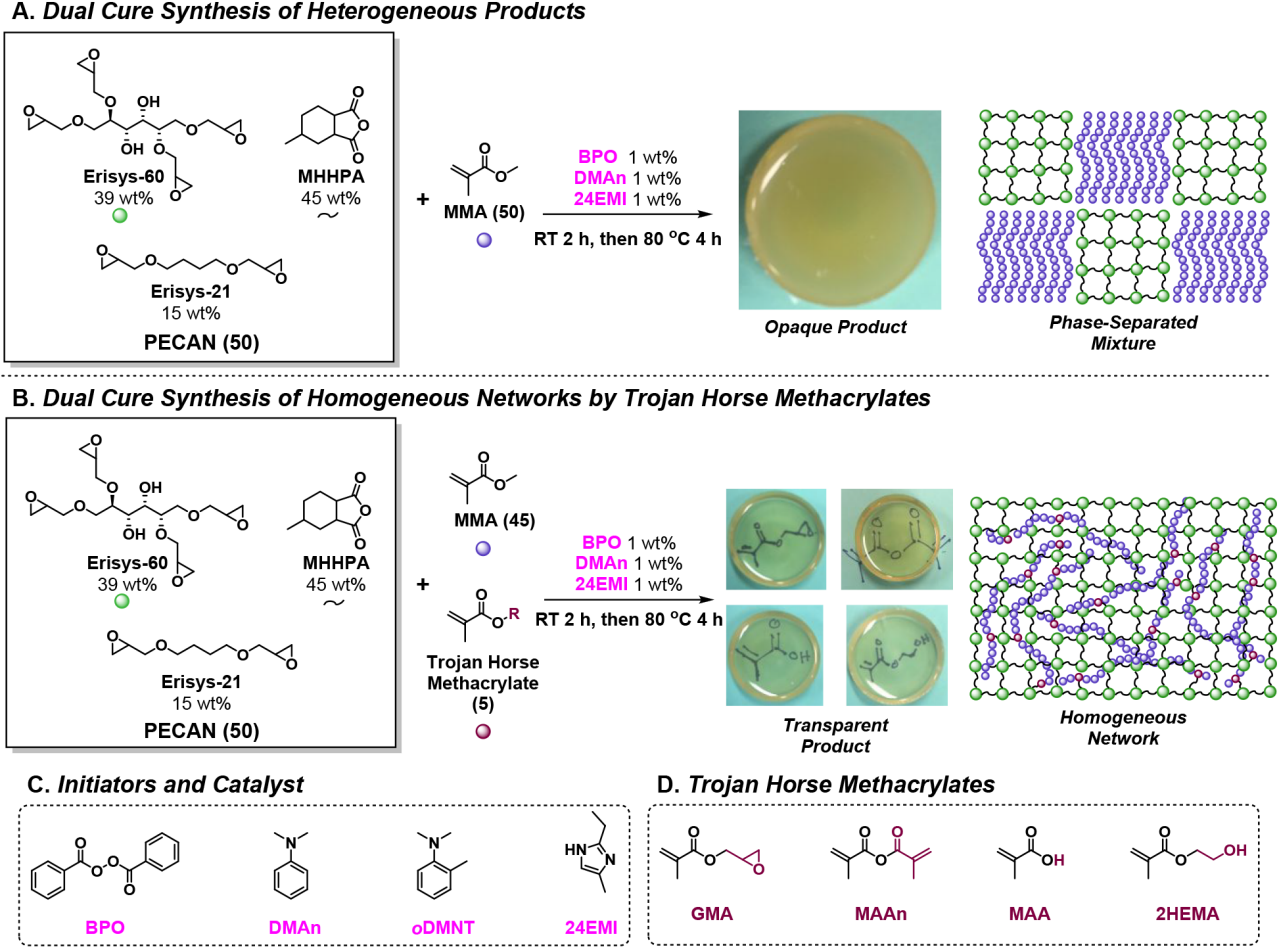
Basic components of DC reactions. A general schematic of DC polymerizations (A) without and (B) with a TH methacrylate, showing chemical structures for all main components, photographs of some polymer products, and illustrations of hypothesized morphologies, as well as structures of select (C) initiators/catalysts and (D) TH methacrylates. Abbreviations: MMA, methyl methacrylate; MHHPA, methylhexahydrophthalic anhydride; BPO, benzoyl peroxide; DMAn, *N*,*N*-dimethylaniline; oDMNT, *N*,*N*-dimethyl-*o*-toluidine; 24EMI, 2-ethyl-4-methylimidazole; GMA, glycidyl methacrylate; MAAn, methacrylic anhydride; MAA, methacrylic acid; 2HEMA, 2-hydroxyethyl methacrylate.

The PECAN formulation, PECAN-39 (Figure 2A,B), used throughout this study is composed of a bioderived polyfunctionalized sorbitol epoxy (Erisys-60, EEW = 179 g/mol, 39 wt %; note that the structure given in Figure 2 is only a representative monomer, while Erisys-60 is actually a mixture of several similar molecules) for rigidity, butanediol diglycidyl ether (Erisys-21, EEW = 126 g/mol, 15 wt %) as a reactive diluent and for toughness/flexibility, methyl-hexahydro-phthalic anhydride (MHHPA, 45 wt %) as the anhydride hardener, and 2-ethyl-4-methyl imidazole (24EMI, 1 wt %) as the initiator/catalyst (Figure 2C). This particular formulation employed a rather large stoichiometric excess of epoxide (∼30 mol % relative to anhydride) to ensure a sufficient epoxide concentration to satisfy reactions with the TH methacrylate (*vide infra*). In other words, instead of reformulating PECAN for each variation on the methacrylate side, we chose one formulation with an excess of reactive epoxides to accommodate all of the methacrylate variants. However, in order to avoid stoichiometric imbalances complicating the thermomechanical analysis, we opted to dial in the stoichiometry for thermomechanical experiments by varying the MHHPA concentration.

As mentioned above, methacrylate monomers were chosen since they are commercially available, structurally and functionally diverse, compatible with various free radical polymerization methods, and generate a substantial heat of polymerization (Δ*H*_p_). MMA was used primarily in this study as it is the simplest and most broadly available methacrylate. EMA was used as a secondary example to demonstrate the effects of both a softer and lower *T*_g_ methacrylate polymer while providing a less volatile monomer when experiments demanded. Other methacrylates, mixtures of methacrylates, or even more reactive acrylate monomers could be used to modulate mechanical properties and/or Δ*H*_p_ but were not employed here. GMA, MAAn, MAA, and 2HEMA were used as TH methacrylates and shown to work, but MAA was chosen to be the focus of this study (Figure 2D). We used a benzoyl peroxide/dimethylaniline (BPO/DMAn, Figure 2C) redox initiation system^37^ for the majority of this work because it allows for RT initiation; however, we also used azobis(isobutyronitrile) (AIBN) as a radical initiator where it was necessary for fundamental studies or differential scanning calorimetry experiments.

We started this investigation by mixing 1 g of PECAN-39 with 1 g of MMA in a 20 mL scintillation vial. This reaction will be referred to as PECAN(50)–PMMA(50) as the numbers in the parentheses represent the weight % of the monomer components. Then, after adding 0.010 g of 24EMI and 0.010 g of BPO, the scintillation vial was attached to a Firestone valve where several consecutive vacuum/N_2_ purge cycles were applied. Finally, 0.010 g of DMAn was added to start the reaction at RT. The reaction was gently shaken to mix the DMAn and then allowed to react at RT for 2 h, at which point the reaction was a semihard gel. These small 2 g scale uninsulated reactions generally do not get hot enough to completely cure the PECAN. Therefore, an oven was used at 80 °C for an additional 4 h and 160 °C for 1 h to complete the cure. The result was an opaque, cream-colored solid (Figure 2A). Four very similar reactions were then executed, the difference being that 5 wt % of a selected TH monomer was substituted in for a corresponding amount of MMA. These four reactions [PECAN(50)–PMMA(45)–GMA(5); PECAN(50)–PMMA(45)–MAAn(5); PECAN(50)–PMMA(45)–MAA(5); PECAN(50)–PMMA(45)–2HEMA(5)] produced hard yellow solids that were now transparent (Figure 2B).

Importantly, when a reaction is designed for chemical heating, it is imperative to consider heat transfer and the effects of scale and insulation. For example, a 200 g scale reaction that reaches a peak temperature of 200 °C in a well-insulated system might only reach 60 °C if run on the 2 g scale. Therefore, in order to prevent having to run every experiment on the 200 g scale, we opted to use a 2 g scale for most reactions, in which case an oven postcure schedule of 80 °C for 4 h followed by 160 °C for 1 h was employed. While we have independent experiments (*vide infra*) meant to characterize the chemically heated sample with respect to oven-heated samples, it is critical to bear in mind that differences in the thermal history of polymers (*i.e.*, differences between chemically heated and oven-heated samples) may impart small differences to the final material.

### Thermodynamics and Calorimetry

**Figure 3:**
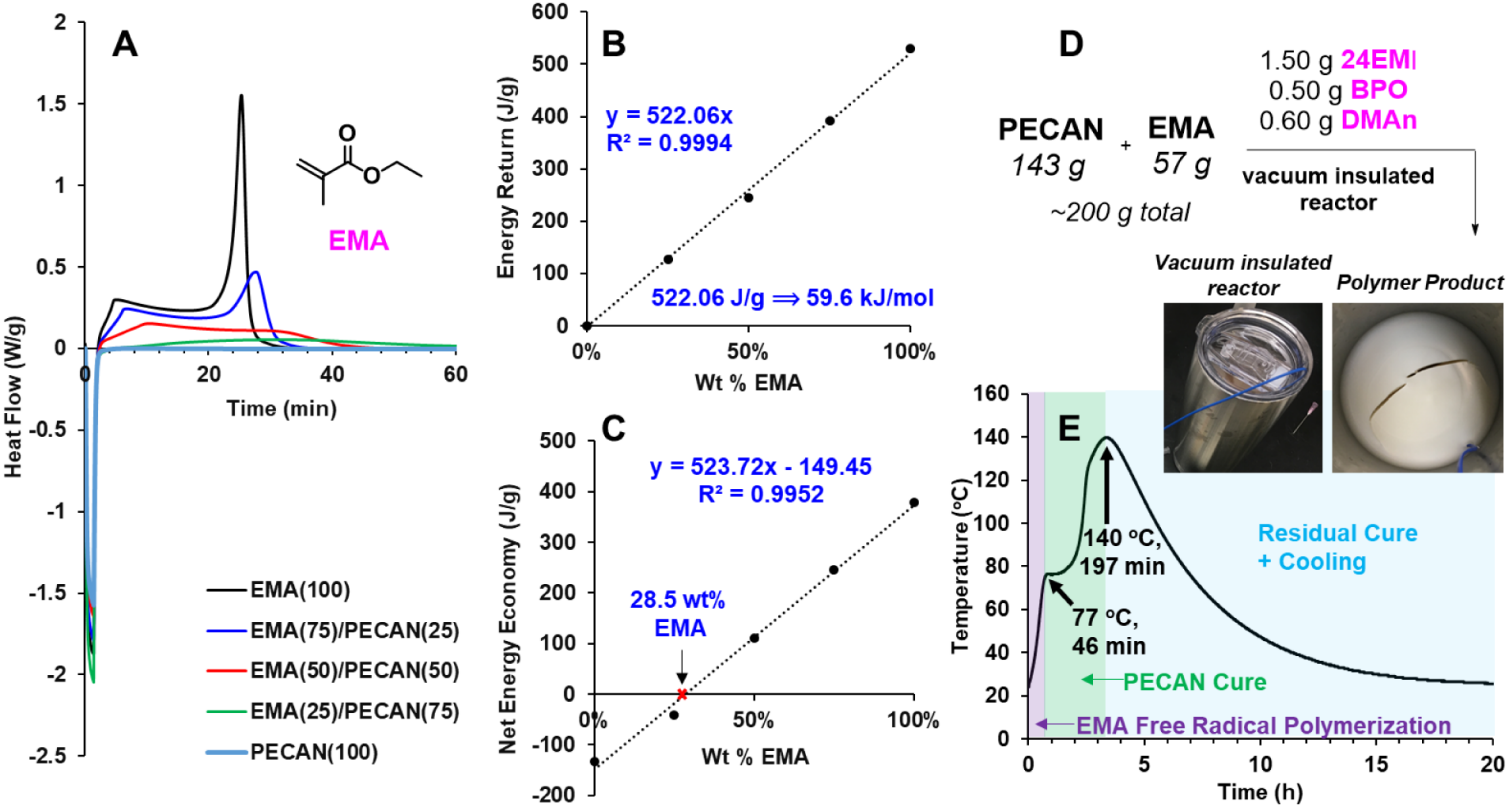
Results of thermodynamics and calorimetry experiments. (A) DSC isothermal temperature *vs* time plot of EMA polymerizations diluted in different amounts of PECAN at 80 °C, *with no PECAN catalyst present*. (B) Energy return *vs* wt % EMA plot; *i.e.*, the integration of only the positive values from (A), which shows isolated thermodynamics of EMA polymerization at different concentrations. (C) Net energy economy *vs* wt % EMA plot, *i.e.*, the integration of all values from (A), to compare the energy cost of heating to 80 °C from RT to the energy provided by the EMA polymerization. (D) Reaction conditions involved in our 200 g scale PECAN(71.5)–PEMA(28.5) calorimetry experiment, which are based on the conclusions inferred by (C), as well as photographs of the reactor and polymer product. (E) Temperature *vsersuss* time profile for the calorimetry experiment described in (D), annotated to communicate the chemical heating proof of concept. Abbreviations: EMA, ethyl methacrylate; 24EMI, 2-ethyl-4-methylimidazole; BPO, benzoyl peroxide; DMAn, *N*,*N*-dimethylaniline.

Following our demonstration of the polymerizability of these polymers, we wanted to investigate the thermodynamic principles of the DC system. The heat generated from a perfectly insulated DC system should respond linearly to a change in the methacrylate content. Therefore, we ran a suite of dynamic scanning calorimetry (DSC) experiments to test this linear relationship between the energy return (*i.e.*, the energy produced from the methacrylate exotherm) and the methacrylate content. We employed EMA for these experiments to avoid volatility issues and omitted 24EMI (or any free base) to exclude any exotherm signal associated with PECAN polymerization. Likewise, to prevent any polymerization prior to data collection, we used AIBN as the thermal radical initiator, which would only thermally initiate once heat was applied. PECAN(*X*)–PEMA(*Y*) reactions were prepared the same way for five different ratios [PECAN(100); PECAN(75)–PEMA(25); PECAN(50)–PEMA(50); PECAN(25)–PEMA(75); PEMA(100)] and deposited into DSC pans. The DSC regimen was an 80 °C isotherm for 80 min as 80 °C will activate AIBN for free radical polymerization. Thus, with the absence of a PECAN catalyst, we can integrate the associated peaks for their thermal energy contribution and neglect the thermal contribution of PECAN polymerization, since no basic catalyst is present. This assumption is reinforced by the fact that the PECAN(100) run yielded almost no exotherm (Figure 3A).

Figure 3A shows the DSC isotherm traces for the five different formulations. The energy return values (Figure 3B) are integrations of the positive values. These integrations, when normalized for the moles of EMA present in each run, correlate linearly and provide a slope value *vs* EMA wt % that corresponds to EMA’s theoretical Δ*H*_p_ of 59.0 kJ/mol.^36^ The negative heat flow values on the DSC trace represent the energy put into the reaction in order to heat from RT to 80 °C. Thus, integration of the entire trace yields the net energy economy—the energy return minus the energy cost of heating to 80 °C—for each formulation (Figure 3C). A good correlation (*R*^2^ = 0.9952) is received, which allows us to predict that 28.5 wt % EMA in PECAN should heat the reaction to 80 °C from RT. Additionally, the spike in enthalpy seen in PEMA(100) and PECAN(25)–PEMA(75) known as the Trommsdorff effect,^34^ is greatly diminished with increasing PECAN composition.

While thermodynamics dictates the amount of thermal energy released by the methacrylate polymerization, the actual temperature that any reaction achieves is governed in part by the heat transfer to its surroundings. Thus, the size/shape of the reaction vessel, the surface area/volume ratio, and the amount of insulation will greatly affect the peak reaction temperature. We wanted to test our prediction from the net energy economy calculation that 28.5 wt % EMA in PECAN should be sufficient to heat the reaction from RT to 80 °C and formulated a PECAN(71.5)–PEMA(28.5) reaction at a 200 g scale (Figure 3D). In order to make this reaction as adiabatic as possible, we used a vacuum-insulated reactor (VIR) to minimize the thermal energy escaping the system. This reaction was prepared by mixing PECAN with methacrylate components as well as initiators BPO and 24EMI in a Schlenk flask connected to a Firestone valve. After several vacuum/N_2_ cycles, the ∼200 g mixture was poured into a VIR. DMAn was injected *via* syringe to react with the BPO to generate free radicals at RT, and a blanket of argon gas was poured into the headspace to minimize O_2_ contamination. A thermocouple probe was dipped into the center of the reaction, and the reactor was then covered. The temperature *vs* time profile (Figure 3E) shows a two-stage heating profile wherein an increase in temperature is observed from RT to 77 °C during the first 46 min representing the EMA free radical polymerization phase. After 46 min, the temperature ramp paused for a few minutes before rising to a peak of 140 °C at 197 min, representing the PECAN polymerization phase. While the reaction fell short of the predicted 80 °C, this is likely because our adiabatic VIR is not perfectly adiabatic. Nonetheless, this reaction (Figure 3E) represents a successful proof of concept of the chemical heating method. It can be seen from Figure 3E that the methacrylate free radical polymerization heats the reaction to a necessary temperature (77 °C), at which point the PECAN reaction can initiate and polymerize, sustained by its own exotherm up to high temperatures to achieve a high degree of cure without any external heating elements. Figure S2 provides additional examples of large-scale DC thermograms.

### Morphology

Now, with a proof of concept in hand, we investigated the DC material in terms of morphology. The extreme difference in character between DC polymers with and without TH monomers (transparent Figure 2B, opaque Figure 2A, respectively) both visually and mechanically (*vide infra*) implies morphologic differences. Since structurally disparate polymers are rarely miscible, it follows that free-flowing PMMA would separate out of the PECAN mixture to form its own domain. The TH strategy, which covalently binds the PMMA chains into the PECAN network during polymer growth, should not allow for this domain separation.

To test this hypothesis, we soaked a sample of PECAN(50)–PMMA(50) in deuterated chloroform overnight. The following day, the sample changed to a milky white suspension. After filtration through a 0.5 μm syringe filter into an NMR tube, the sample was sealed and sent for H NMR analysis. The obtained spectrum clearly showed peaks only for PMMA (Figure S3). When an identical experiment was performed on PECAN(50)–PMMA(45)–PMAA(5), no peaks were observed for PMMA or any appreciable other compounds (Figure S4). This result implies that methacrylate chains within the PECAN(50)–PMMA(45)–PMAA(5) structure are all bound into the PECAN network and thus cannot be extracted into solution. Figure S4 includes a photograph of this sample soaked in chloroform for 72 h, totally unaffected by the solvent.

**Figure 4:**
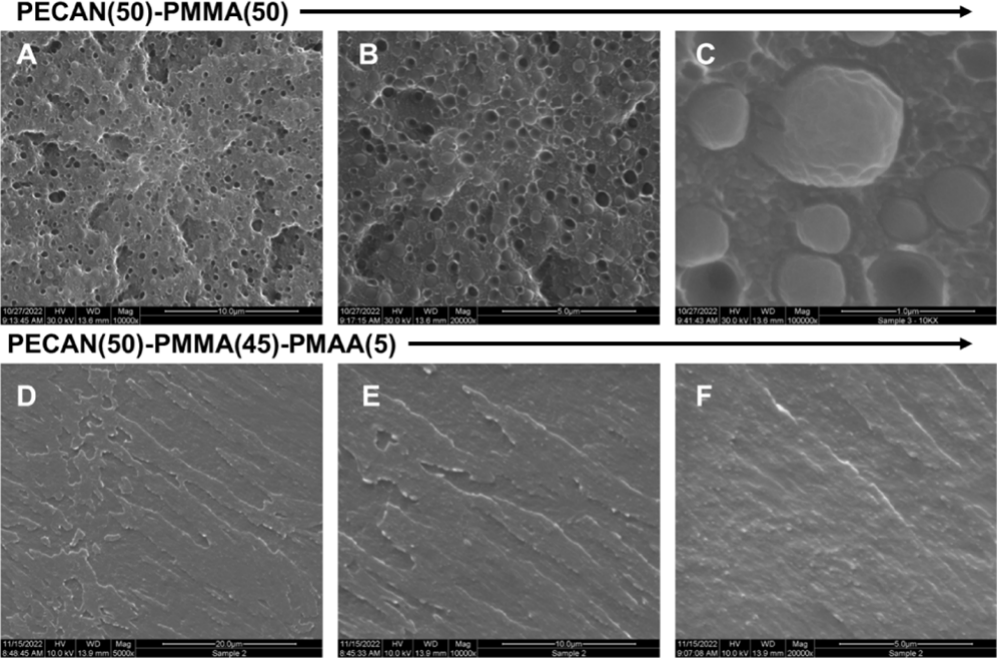
SEM images of selected DC materials. (Top row) PECAN(50)–PMMA(50) at (A) 10,000×, (B) 20,000×, and (C) 100,000× magnification and (bottom row) PECAN(50)–PMMA(45)–PMAA(5) at (D) 5000×, (E) 10,000×, and (F) 20,000×.

Next, we performed scanning electron microscopy (SEM) on a PECAN(50)–PMMA(50) thin film after cryofracture in liquid N_2_. The SEM images (Figure 4A–C) of the fracture surface revealed a heterogeneous structure with spheres (presumably PMMA domains), about 0.5–1 μm in size, suspended in a presumably continuous PECAN phase. Similarly, we obtained SEM images of a PECAN(50)–PMMA(45)–PMAA(5) cryofractured thin film. A smooth, continuous, and homogeneous material was observed with no visible domain separation (Figure 4D–F). Unfortunately, magnification beyond 20,000× was not feasible due to the material’s sensitivity to and degradation by the electron beam.

**Figure 5:**
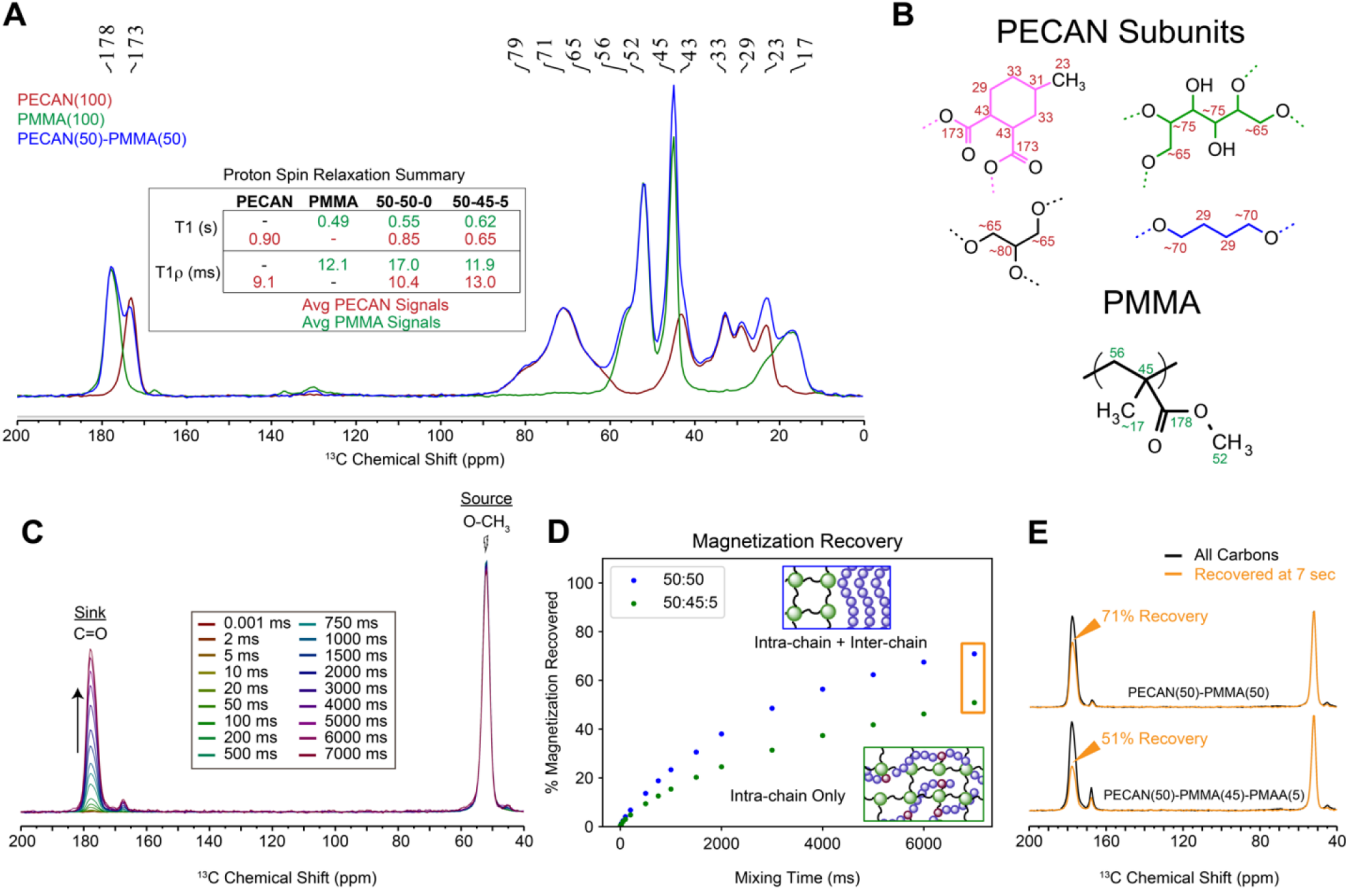
Solid-state NMR probes for nanometer-scale morphology of DC materials. (A) ^13^C CP-MAS spectra of neat PECAN(100) (red/maroon) and PMMA(100) (green) are compared with phase-separated thermoset PECAN(50)–PMMA(50) (blue), at natural abundance. Chemical shifts of key features are indicated. A summary of proton spin relaxation results from Tables S1 and S2 is presented in the table inset. (B) Representative structures of PECAN and PMMA subunits with approximate chemical shift assignments. (C) Stacked plot of selective 1D ^13^C–^13^C spin-diffusion spectra of PECAN(50)–PMMA(50), in which the PMMA component was ^13^C enriched (33%) at either the O*C*H_3_ or the *C*OO sites at spin-diffusion mixing times ranging from 0.001 to 7000 ms. Spectra are normalized to the intensity of the selected (O*C*H_3_) signal to better visualize the ^13^C magnetization between the source (O*C*H_3_) and sink (*C*OO) carbons. (D) Fraction of PMMA ^13^C sink carbons (*C*OO) that have gained ^13^C magnetization from the selected ^13^C source (O*C*H_3_) carbons at each spin-diffusion mixing time for PECAN(50)–PMMA(50) and PECAN(50)–PMMA(45)–PMAA(5) systems. (E) Example at 7000 ms spin diffusion of how each point in (D) is generated. The decrease in recovered *C*OO signal for the PECAN(50)–PMMA(45)–PMAA(5) system compared to PECAN(50)–PMMA(50) is due to the absence of interchain spatial contacts on the subnanometer length scale.

However, as it is possible that phase separation still occurs in smaller domains beyond our capable magnification, we conducted a complementary solid-state NMR (ssNMR) study to probe polymer miscibility. Proton spin relaxation rates in both the laboratory (^1^H T_1_) and rotating frames (^1^H T_1ρ_) in the solid state are sensitive to nanoscale separation of domains; an averaging of ^1^H relaxation rates due to efficient ^1^H–^1^H spin-diffusion between domains is indicative of polymer miscibility over the length scales defined by the experiment.^99^Figure 5A shows an overlay of ^13^C CP-MAS spectra of neat PECAN, PMMA, and PECAN(50)–PMMA(50)–PMAA(0), at natural ^13^C abundance. The PECAN(50)–PMMA(45)–PMAA(5) spectrum is omitted for clarity. General ^13^C assignments and structural motifs are provided in Figure 5B. To investigate polymer miscibility on the tens of nanometer and 2–3 nm length-scales, we analyzed ^1^H T_1_ and T_1ρ_ relaxation rates, respectively. We clearly observed T_1_ and T_1ρ_ averaging for the PECAN(50)–PMMA(45)–PMAA(5) “homogeneous” sample but insignificant averaging for the phase-separated PECAN(50)–PMMA(50) sample (Figure 5A inset, Tables S1 and S2). This suggests PECAN(50)–PMMA(45)–PMAA(5) is homogeneously mixed, at least on the 2–3 nm scale, but domain separation is much larger than ∼50 nm when the TH is not included. Results are further described in the Supporting Information.

Since the above results cannot distinguish between complete mixing and the presence of small (∼2–3 nm) PMMA/PECAN nanodomains, we next turned to ^13^C–^13^C spin-diffusion methods to further investigate if chain mixing occurs on the subnanometer level. We prepared PECAN(50)–PMMA(50) and PECAN(50)–PMMA(45)–PMAA(5), in which PMMA was polymerized from the following blend: 1/3 MMA with ^13^C-enrichment at O*C*H_3_, 1/3 MMA with ^13^C-enrichment at *C*OO, and 1/3 MMA at natural abundance. Thus, every methacrylate unit is either unlabeled (33.3%), ^13^C-labeled at O*C*H_3_ only (33.3%), or ^13^C-labeled at *C*OO only (33.3%). We then applied a selective 1D ^13^C–^13^C spin-diffusion technique to probe ^13^C–^13^C spatial interactions,^100,101^ which are sensitive up to ∼0.8–1 nm.^102^ Our approach involves first selecting a resolved ^13^C signal to generate isolated ^13^C magnetization (source), and then monitoring the time-dependent equilibration of ^13^C signal as it spreads from the selected source to proximal ^13^C sites (sinks). Longer spin-diffusion mixing periods correspond with longer intercarbon distances, with a 1/*r*^6^ relation.^103^ In our case, the source is the selected O*C*H_3_ signal at 52 ppm and the sink carbons are ^13^C *C*OO sites.

The important observable is to quantify the percentage of sink carbons that reside within the spin diffusion range of source carbons at each spin-diffusion mixing time. The technique is sensitive up to about 1 nm at the longest mixing times.^102,104^ The core concept is that an isolated, linearized PMMA chain should only show intrachain ^13^C–^13^C interactions, for example, O*CH*_3_(*n*) to *C*OO(n ± 1, n ± 2), but stacked PMMA chains in a phase-separated domain should have additional ^13^C–^13^C contacts between O*C*H_3_ of chain A and *C*OO of chain B. We hypothesized based on the proposed morphologies that phase-separated PECAN(50)–PMMA(50) material should show both intrachain ^13^C–^13^C through-space contacts and also interchain interactions due to the tight packing of phase-separated PMMA. For the TH system [PECAN(50)–PMMA(45)–PMAA(5)], we hypothesized that if polymer mixing within PECAN is homogeneous such that multinanometer PMMA stacking is prevented, intrachain^13^C–^13^C contacts would dominate while interchain contacts would be sparse or absent.

Figure 5C–E shows magnetization recovery data for ^13^C–*C*OO carbons (sink) as they receive magnetization from selected ^13^C–O*C*H_3_ (source) sites during a variable spin-diffusion mixing period τ_m_ from very short (0.001 ms) to very long (7000 ms). The PECAN(50)–PMMA(45)–PMAA(5) shows substantially reduced O*C*H_3_–*C*OO spatial interactions compared to the phase-separated PECAN(50)–PMMA(50) material in which large PMMA domains are present. Together, ^1^H–^1^H and ^13^C–^13^C spin-diffusion results support the proposed morphologies that PECAN(50)–PMMA(50) and other materials not containing a TH methacrylate are phase-separated with large (>500 nm) domain sizes. Importantly, the PECAN(50)–PMMA(45)–PMAA(5) and other materials containing a TH methacrylate are homogeneously mixed at the subnanometer length scale.

### Thermomechanical Properties

**Figure 6:**
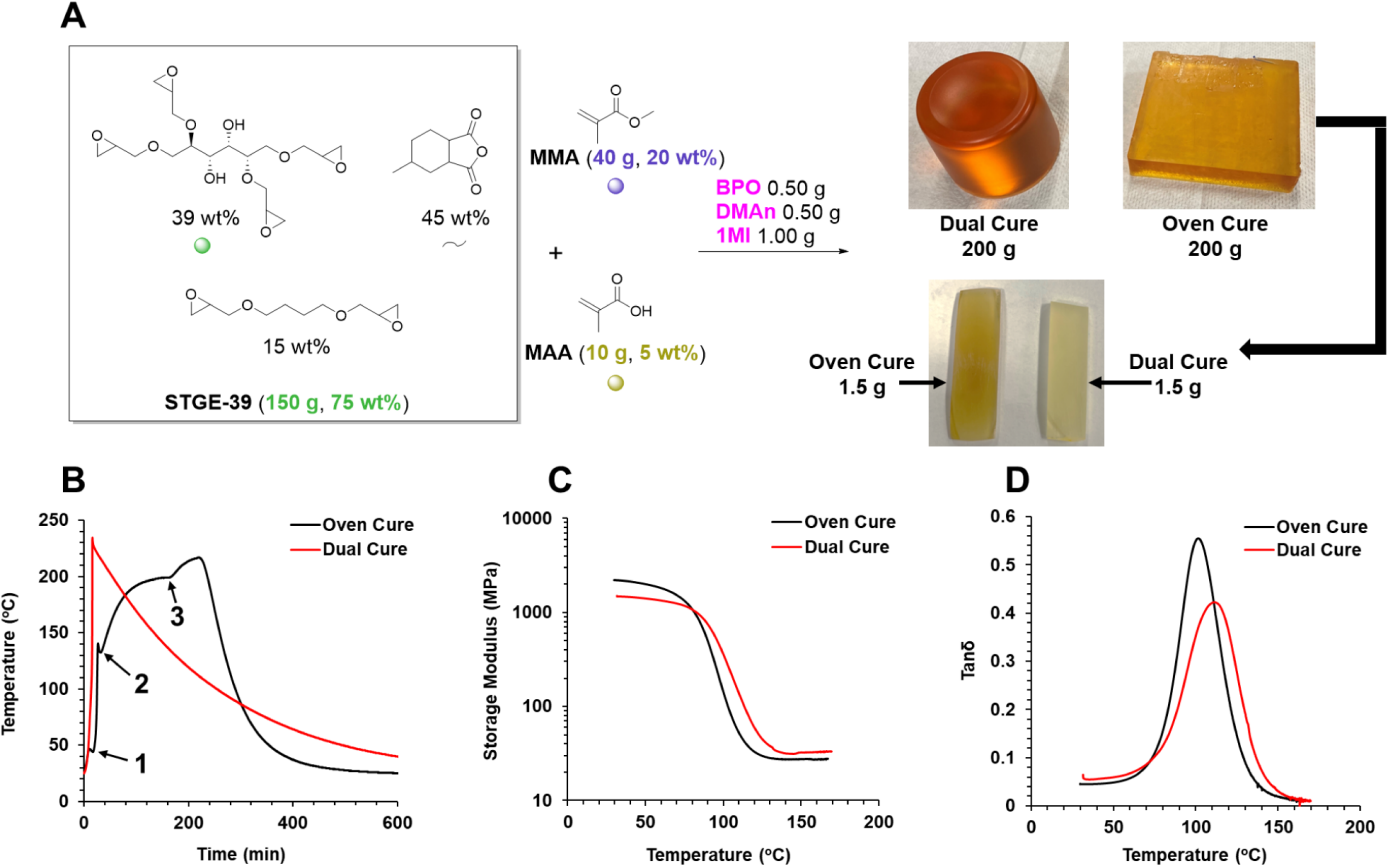
Dual cure *vs* oven cure characterization of PECAN(75)–PMMA(20)–PMAA(5) materials. (A) Synthesis of both 200 g scale oven cure and dual cure samples, where dual cure means only chemical heating was employed and no supplemental heat was added, (B) thermogram of oven cure *vs* DC heating schedules, where DC involves only chemical heat in a well-insulated reactor to achieve a peak temperature of 230 °C while oven cure involves curing in a noninsulated mold and allowing the temperature to peak at 47 °C from the methacrylate exotherm (1) before placing the reaction in an oven preheated to 230 °C where the temperature quickly spiked (2) to 144 °C due to the PECAN exotherm and then slowly increased to 200 °C from oven heat, at which point the oven temperature was then increased (3) to 250 °C causing the reaction temperature to slowly increase to 216 °C. Additionally, (C) DMA storage modulus *vs* temperature plot of oven cure *vs* DC samples and (D) tan δ *vs* temperature plot for oven cure *vs* DC samples.

Next, we wanted to test the DC materials prepared solely by chemical heating, with no prior postcure treatment, and compare this result to an analogous oven-cured sample. Since any DC reaction is subject to heat transfer and loss of chemical heat to the environment, we ran these syntheses on a rather large 200 g scale. We prepared a 400 g scale reaction, which was later partitioned into two separate 200 g scale reactions. One of the 200 g scale reactions was executed in a well-insulated VIR, while the other was executed in a noninsulated mold. We used the same PECAN-39 formulation in a PECAN(75)–PMMA(20)–PMAA(5) weight ratio as the 5 wt % MAA makes for balanced stoichiometry assuming 1 epoxide will react with one anhydride and one epoxide will react with one carboxylic acid (Figure 6). To maximize the peak temperature, we employed the more highly active PECAN initiator/catalyst 1-methyl imidazole (1MI). This 1MI catalyst was expected to accelerate PECAN initiation and cure, allowing for peak temperatures to be reached in a shorter time frame so that there would be less time spent dissipating heat, greater peak temperatures, and faster PECAN reactivity during the time spent at high temperatures. A dramatic increase in peak temperature to 230 °C at 17 min can be observed for the DC sample in Figure 6B. While we attempted to provide a comparable thermal history for the oven-cured sample by first allowing the methacrylate exotherm to dissipate outside the oven (reaching a max temperature of 47 °C) and then placing the sample in an oven preheated to 230 °C, we found the heating rate in the oven to be extremely sluggish compared to chemical heating. Thus, after the PECAN exotherm and 2 h in the 230 °C oven, a peak temperature of only 200 °C was reached. Attempting to get this reaction to the analogous 230 °C, we increased the oven temperature to 250 °C. However, it became evident that the temperature was going to peak around 216 °C so we shut the oven off at 228 min to avoid overexposure and oxidation of the material.

These 200 g samples were again machined down to 1.5 g of DMA coupons and subjected to DMA temperature sweeps (Figure 6C,D). While somewhat closer to the expected *T*_g_, the materials differed substantially in storage moduli and tan δ with the oven-cured sample having a storage modulus of 2241 MPa *vs* the DC sample measuring 1477 MPa at 35 °C. Additionally, the DC sample expressed a *T*_g_ of 111 °C, while the oven-cured sample had a *T*_g_ of 101 °C. Both samples expressed a similar plateau modulus of 170 MPa. DMA traces of the second and third cycles, where the thermal history for both samples is neutralized, reveal persistent differences in the two materials (Figure S5). It is unclear whether these differences are due to differences in the heating schedule and thermal history or if the oven-cured sample was damaged by oxidation (the coupon itself had a brownish tint compared to the normal yellow, Figure 6A). Nonetheless, the extent of cure and differences in thermal history will always affect the thermomechanical character of a material and need to be accounted for when designing a DC reaction, especially when no supplemental heat is provided.

Next, we wanted to compare DC materials to their parent PECAN material in terms of thermomechanical properties and determine the effect of TH content on the thermomechanical properties. To do this, we reformulated the PECAN-39 formulation used throughout this study to have balanced stoichiometry by keeping the Erisys-21 and Erisys-60 ratio the same but slightly increasing the MHHPA content (as we do not want an excess of unreacted epoxides present during the thermomechanical tests). We then formulated four different DC samples with increasing TH content. Since TH presumably reacts with epoxide (Figure 1), the PECAN components had to be slightly rebalanced for each formulation to ensure even stoichiometry (Table 1). This does make it hard to eliminate variables since the PECAN formulation is slightly different in each run. However, the variables kept constant were the Erisys-21/Erisys-60 weight ratio (1/2.45) as well as the PECAN/total methacrylate weight ratio (3/1). These samples were prepared at a 10 g scale and employed 1MI as the PECAN catalyst at 1 wt % of the PECAN weight as well as BPO and DMAn at 1 wt % of the total methacrylate weight. These 10 g reactions were degassed *via* several vacuum/N_2_ cycles before initiation by the addition of DMAn. They were then partitioned into 2 g of samples by pouring into 20 mL polypropylene vials. The vials were purged with Ar gas and sealed, then laid on their side, and left to react for 1 h. At this point, they had gelled due to the methacrylate polymerization, but the PECAN monomers were still largely uncured. We then heated them in an oven at 80 °C for 8 h causing them to solidify. Finally, after each sample was removed from its container, each sample was machined down into uniform dimensions to yield DMA coupons of 1.5 g.

**Table 1:**
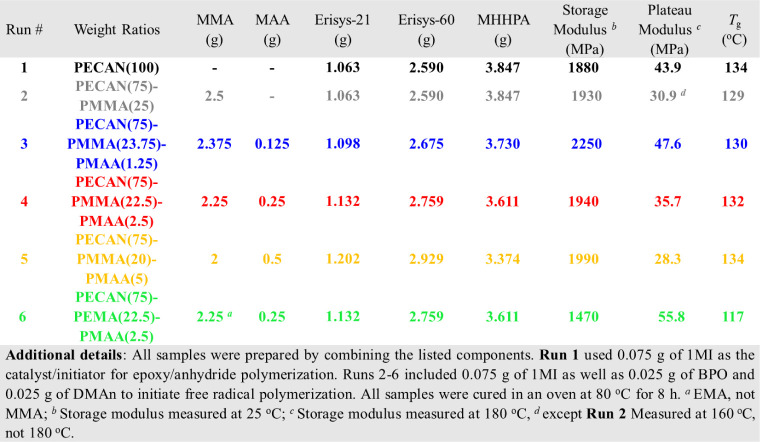
Specific Formulations Used for DMA Studies

**Figure 7:**
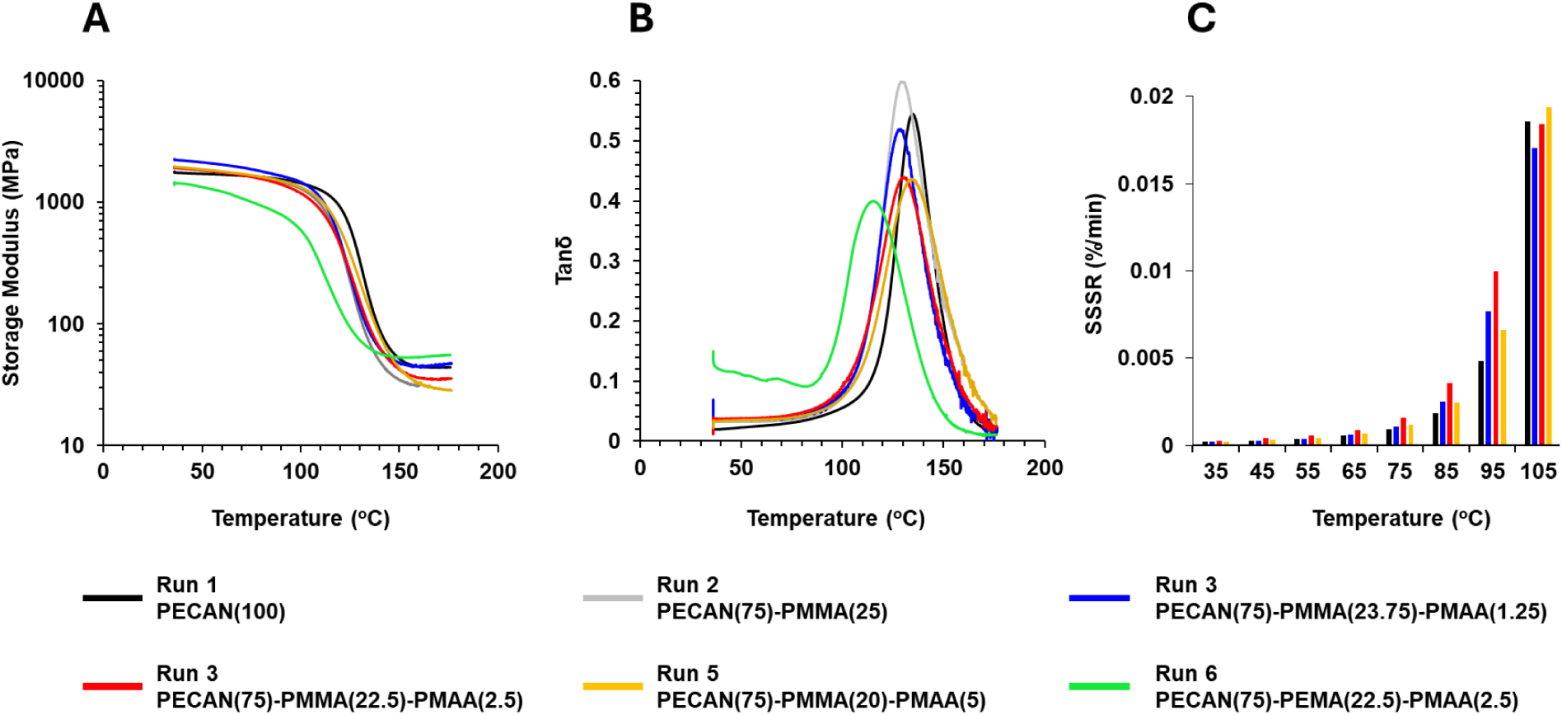
DMA and creep characterization of DC *vs* PECAN materials. Run #s correspond to formulations found in Table 1. (A) Storage modulus *vs* temperature profiles for parent PECAN *vs* DC materials with varying TH content, (B) tan δ *vs* temperature profiles for parent PECAN *vs* DC materials with varying TH content, and (C) creep *vs* temperature as determined by the steady-state strain rate (SSSR) of parent PECAN *vs* selected DC materials with varying TH content. See Figure S9 for the full creep data set. All DMA and creep data shown in this figure represent materials that were pretreated with a prior heating cycle intended to normalize thermal history.

Again, 80 °C for 8 h is not sufficient for complete and total cure, and small differences in thermal history will alter the thermomechanical character of the material. Thus, to eliminate variables, we used the DMA temperature sweep itself to complete the cure for each sample during the first cycle. Following the first temperature sweep up to 180 °C, the sample was allowed to slowly cool back down to 35 °C. This was repeated twice, with the second and third cycles exhibiting storage moduli and tan δ profiles identical to each other but differing from the first cycle (Figures S6 −and S7). This indicates that the cure was incomplete during the first cycle but complete during the second cycle. Thus, all materials were compared on the basis of the second DMA cycle, as shown in Figure 7A,B.

Gratifyingly, the DC materials employing MMA and MAA performed similarly to their parent PECAN system, with only minor differences in their DMA profiles. Most notably, the tan δ peaks were broader for all DC samples and their onset *T*_g_ was slightly lower. This is likely due to the incorporation of PMMA which has a lower *T*_g_ (∼120 °C) than that of the parent PECAN (135 °C). Additionally, the broadness of tan δ might be caused by an increase in hydroxyl groups formed by the epoxy/carboxylic acid reaction (Figure 1), which acts as a dynamic exchange agent for transesterification throughout the network. Interestingly, the plateau modulus slightly decreased with increasing TH content, which was not expected as the cross-link density should be determined by the epoxide content, which was higher for those formulations with higher TH. Similarly, the storage modulus at 35 °C decreased with increasing TH content with Run 3 having a modulus of 2250 MPa and Run 5 having a storage modulus of 1990 MPa, while the parent PECAN had a modulus of 1880 MPa. The *T*_g_ of the DC materials increases with TH content with Run 3 having a *T*_g_ of 130 °C while Run 5 has a *T*_g_ of 134 °C, approaching that of the parent PECAN which has a *T*_g_ of 135 °C. In the absence of TH, Run 2 performed surprisingly well while having only a slightly lower *T*_g_ (129 °C) and plateau modulus (30.9 MPa) than the parent PECAN(100). However, this material was surprisingly brittle and cracked in the DMA around 160 °C. Lastly, when EMA (Tg ∼ 60 °C) is used instead of MMA as the structural methacrylate component, a large drop in storage modulus is observed (1470 MPa as opposed to the analogous Run 4, 1940 MPa) as well as substantial tan δ at lower temperatures (35–75 °C). This implies that there may be some microdomain separation between PECAN/methacrylate phases. The main tan δ peak gives a *T*_g_ of 117 °C, a significant decrease from the parent PECAN, due to the lower *T*_g_ of PEMA. Run 6 does surprisingly give the highest plateau modulus (55.8 MPa) of all the samples tested, for which the authors do not have an explanation. This seemingly strange behavior from Run 6 was reproducible over multiple cycles and is included in Figure S7.

We also performed creep tests by first subjecting the coupons to a DMA temperature sweep to achieve full cure and erasure of thermal history, then soaking them at a temperature for 10 min, then subjecting them to 1 MPa for 30 min, and measuring strain response with respect to time. Temperatures were measured consecutively in 10 °C intervals between 35 and 105 °C (Figure S8). By the end of each 30 min, each sample had reached a steady-state strain rate (SSSR) with respect to time, which we measured in units of % strain/min. The SSSR of each sample at each temperature is shown in Figure 7C. Only subtle differences in creep can be observed between samples, presumably related to minor differences in the *T*_g_. Nonetheless, this result demonstrates that DC materials perform competitively with their parent PECAN analog in terms of creep resistance. Creep data were measured for Runs 2 and 6 and can be seen in Figure S9 but were omitted from Figure 7 for clarity.

### Polymer Deconstruction

The DC materials, being a mixture of polyesters and thermoplastics, are intended to be recyclable at the end-of-life. In the case of glass or carbon-fiber reinforced composite applications, an important option is to degrade the thermoset material in order to isolate and collect the fiber component for a second life, as the fiber component has a much greater environmental and economic impact than the thermoset. Generally, methanolysis can be used to degrade PECAN networks by transesterifying all ester bonds to give methyl esters of the previously anhydride monomers and alcohols of the epoxide monomers. In the case of DC materials, methanolysis can plausibly be employed to yield the same methyl esters and alcohols, as well as PMMA (Figures S10–S12). While this would to some extent complicate product separation, PMMA being insoluble in methanol should simply precipitate out of the depolymerization mixture.

**Figure 8:**
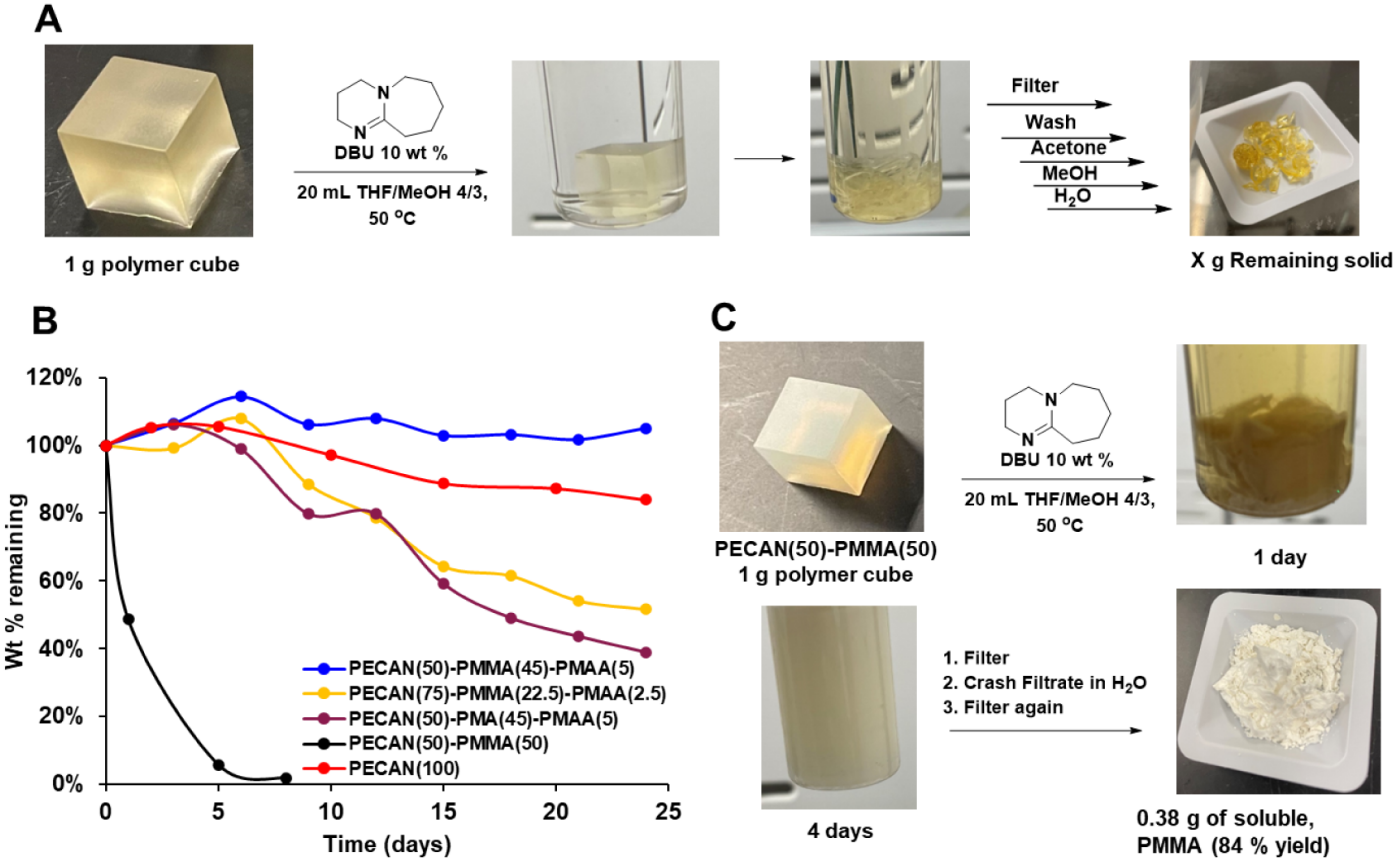
Results of polymer deconstruction/methanolysis studies. (A) Standard methanolysis conditions to which each cube was subjected to. (B) Degradation *vs* time plot for five different formulations. (C) Photos of the PECAN(50)–PMMA(50) runs and recovered PMMA. Abbreviations: DBU, 1,8-diazabicyclo(5.4.0)undec-7-ene; PMA, poly(methyl acrylate).

However, we found experimentally that deconstruction is much more difficult for DC materials than for the parent PECAN material. The methanolysis strategy used in previous work^62^ employs pure methanol with K_2_CO_3_ to deconstruct PECAN or with a cosolvent such as dichloromethane or acetone.^105^ Here, we found that DC samples require a cosolvent to achieve any breakdown at all, possibly due to the complex nature of the polymer. We suspect that the insolubility of PMMA in methanol prevents any solvent penetration into the material under the deconstruction conditions employed. After several unsuccessful attempts to deconstruct PECAN(50)–PMMA(45)–PMAA(5), we settled on what we thought to be a logical set of conditions to compare a few formulations and to explore important parameters. For this, 1 g cubes of polymer from five different formulations [PECAN(100), PECAN(50)–PMMA(45)–PMAA(5), PECAN(75)–PMMA(22.5)–PMAA(2.5), PECAN(50)–PMA(45)–PAA(5), and PECAN(50)–PMMA(50)] were synthesized (PMA is poly(methyl acrylate)). Ten vials of each formulation were prepared containing one 1 g polymer cube, 20 mL of solvent [12 mL of tetrahydrofuran (THF) and 8 mL of methanol), and 100 mg of 1,8-diazabicyclo(5.4.0)undec-7-ene (DBU) (10 wt % relative to polymer). The use of THF was intended to help solubilize the methacrylate polymer. Each vial was equipped with a “flea”-sized stir bar and heated to 50 °C with stirring. Every 2–5 days, a sample would be removed from the heating block, filtered over a fine frit, washed with acetone, methanol, and water, and then dried for 24 h at 80 °C under a vacuum (Figure 8A). Finally, the solid product was weighed and tabulated as wt % of the original cube (Figure 8B). Important to note is that every time point in Figure 8B is an individual cube.

The PECAN(100) sample plateaus around 87 wt % after approximately 20 days, in good agreement with our past studies,^62^ due to Le Chatelier limitations wherein the methanol is likely becoming saturated with degradation products. In contrast, PECAN(50)–PMMA(45)–PMAA(5) actually increases in weight and never gravimetrically degrades at all. We suspect the increase in weight is due to methanolysis, which only adds methanol into the network without producing any soluble fragmentation. Interestingly, reducing the methacrylate content to 25 wt % [PECAN(75)–PMMA(22.5)–PMAA(2.5)] allows for steady degradation to 52 wt % after 25 days. This result is likely due to a reduction in methacrylic polymer composition, which prevents methanol penetration into the network. Likewise, changing the PMMA/PMAA components to PMA/PAA (poly(methyl acrylate)/poly(acrylic acid)) results in a steady decrease to 39 wt % after 25 days. Again, the more soluble and lower *T*_g_ acrylate compared to methacrylate allows more methanol to infiltrate the polymer network.

Lastly, we found that the PECAN(50)–PMMA(50) cubes fell apart almost immediately under these conditions. This is rather unsurprising considering that the PMMA is not covalently tethered to the PECAN network; thus, its dissolution, facilitated by the majority solvent THF, results in its extraction from the bulk material. After 1 day, the remaining solids recovered after filtration were 49 wt %. We suspected this insoluble fraction to be PECAN fragments, while the filtrate was PMMA. The filtrate was then precipitated into water to give a solid white precipitate that was again filtered and washed with water. This recovered material was then weighed and identified to be 0.38 g (84% yield) of soluble PMMA by ^1^H NMR (Figures 8C and S13).

## Discussion

The DC technology put forth in this work attempts to find a middle ground between two extremes. One extreme is PECAN technology, which is biobased, degradable, and offers high-performance cross-linked material at the cost of high energy for heating. The other extreme is methacrylate-based technologies such as Elium which enjoy the ease and convenience of room temperature cure as well as simplified end-of-life options at the cost of using petroleum-sourced carbon and weakened mechanical performance. Our justification for blending the two extremes into some middle ground is to attempt to maximize the advantages of each system while minimizing the disadvantages. In other words, we want to maximize the quantity of the biobased monomer and high mechanical performance while maintaining the energy efficiency and convenience of a room temperature cure. The main drawback of blending these two technologies is that it does complicate the end-of-life process. However, we contend that the methanolysis of the DC material theoretically results in PECAN degradation products and PMMA. Given that PMMA is not soluble in methanol, separation of PMMA from the PECAN degradation products is feasible. While we did not by any means optimize the process in this work, we do not suspect that this additional complexity added by the hybrid material is necessarily prohibitive for the technology.

The main motivation of this work was to cure epoxy resins by chemical heating as opposed to conventional heating (ovens, heated molds, *etc.*). To that end, we have shown a proof of concept. Figure 3D,E shows the successful cure of PECAN/methacrylate resins without the aid of an external heating element. For real-world applications, control of heat transfer is important. Figure S2 shows how a simple change in the degree of insulation can drastically change the peak temperature of the reaction. A glass beaker *vs* a vacuum-insulated reactor results in a peak temperature difference of 183 °C. Some parameters to consider when designing a DC/chemical heating application are the scale/shape of the object to be cured, the surface area to volume ratio, and the degree of insulation. With that said, there are several handles to dial in the ultimate temperature of the cure such as catalyst activity, composition of methacrylate, and even the thermodynamics of the chosen methacrylate (or acrylate). For example, EMA will provide 0.517 kJ/g of thermal energy, while methyl acrylate would provide 0.982 kJ/g, almost twice as much thermal energy.^36^

In terms of green chemistry and sustainability, we predict that the chemical heating concept of DC could yield higher energy efficiency in manufacturing, especially for the curing of physically large components in wind, automotive, aerospace, and building material technologies. Unlike conventional heating elements that heat a bulk material from the outside-inward, DC heats the material evenly and homogeneously throughout. Based on this nuance, we predict that cure times and cure efficiencies will be substantially improved. Additionally, the chemical heating method can greatly simplify the infrastructure and tools needed for the production of thermosets. For example, instead of requiring a heated mold to cure a thermoset component, where the design parameters are focused on the heating element, a simpler mold that does not require electrical components can be used instead. In this case, design parameters can be focused more on insulation and achieving adiabatic conditions so that the natural exotherm is not wasted and energy efficiency can be truly optimized.

Lastly, we want to address the potential concern that by replacing electrical heating with chemical heating, we are just moving the cost from one step to another. In other words, the cost of heating the reaction is just paid for during the synthesis of the (meth)acrylate as opposed to the synthesis of the thermoset. While this is partially true, the (meth)acrylate industry is mature in its development and produces methacrylates on a massive scale. Comparing methacrylate-based energy to grid energy, we argue that methacrylate produced in an efficient manner on a large scale is easily transportable, easily stored for long periods of time, and easily divisible among several individual reactions, unlike grid electricity. Furthermore, the (meth)acrylate component used for chemical heating displaces an equal weight of the thermoset component when compared with that parent thermoset reaction. Thus, some of the energy cost of the (meth)acrylate is offset by the reciprocal reduction in the thermoset feedstock. Importantly, traditional thermoset monomers (such as epoxides and amines) have a high energy cost themselves (∼75 MJ/kg),^27^ thus this monomer displacement factor is not insignificant. Techno-economic analysis/life cycle analysis of this process is ongoing and will provide an objective assessment of the predicted energy savings.

## Data Availability

All data used to reach the conclusions of this manuscript, as well as the Materials and Methods, are provided in the main text or Supporting Information. Data may be made available upon request by contacting the corresponding authors directly.

## Supporting Information Available

More information on the materials used in this study, supplemental methods for polymer synthesis and analysis, and additional results, which include numerical values for select figures, pictures of the experimental setups, additional thermomechanical data, and mechanisms for chemical depolymerization (PDF)
