# Supplementary material for: Synergistic Dual-Cure Reactions for the Fabrication of Thermosets by Chemical Heating
Source: ACS Sustain Chem Eng. 2024 Aug 2;12(32):11913–27. doi: 10.1021/acssuschemeng.4c01965 (PMC11323266; doi:10.1021/acssuschemeng.4c01965)
Supplement: Supplementary file 1 — sc4c01965_si_001.pdf [file sc4c01965_si_001.pdf]

Supporting Information for

Synergistic Dual Cure Reactions for the Fabrication of Thermosets by Chemical Heating

Renewable Resources and Enabling Sciences Center, National Renewable Energy Laboratory, Golden CO, 80401 USA

Michael L. McGraw, Bennett Addison, Ryan W. Clarke, Robert D. Allen, Nicholas A. Rorrer*

*Correspondence to:

Robert Allen at robert.allen@nrel.gov; https://orcid.org/0000-0001-9950-103X

Nicholas Rorrer at nicholas.rorrer@nrel.gov; https://orcid.org/0000-0001-9134-5853

Number of pages: 22

Number of tables: 2

Number of figures: 13

Table of Contents

Supplemental Materials and Methods……………………………………………………S3

Materials.

Synthesis of Dual Cure Materials.

Synthesis of ^{13}C-labeled Substrates.

Figure. S1. Purification and Identification of 13C-labeled MMA.

Analytical Methods………………………………………………………………… S5

Supplemental Figures and Tables………………………………………………………S8

Table S1. Proton Spin Lattice relaxation times (^{1}H T_1).

Table S2. ^{1}H Spin-Lattice relaxation times in the rotating frame ($T_{1\rho}$).

Fig. S2. Large Scale Field Tests of PECAN/MMA DC Reactions by Chemical Heating.

Fig. S3. DC Material Extraction Experiment (No Trojan Horse).

Fig. S4. DC Material Extraction Experiment (Trojan Horse).

Fig. S5. DMA plots of Oven Cure vs Dual Cure samples over multiple cycles.

Fig. S6. DMA plots for Run 1 showing all 3 cycles.

Fig. S7. DMA plots for Run 6 showing all 3 cycles.

Fig. S8. An example strain vs time plot corresponding to the creep tests.

Fig. S9. Creep tests for Runs 1-6.

Fig. S10. Infered methanolysis chemistry (carboxylic acid TH).

Fig. S11. Infered methanolysis chemistry (anhydride TH).

Fig. S12. Infered methanolysis chemistry (hydroxy TH).

Fig. S13. Deconstruction of PECAN(50)-PMMA(50) and NMR of Recovered of PMMA.

References……………………………………………………………………………S21

Supplemental Materials and Methods

Materials

Epoxides Erisys-60 and Erisys-21 were purchased from Erisys. MHHPA, MMA, MAA, EMA, MAAn, HEMA, GMA, methacroyl chloride, DMAn, *o*DMNT, 24EMI, and AIBN were purchased from Sigma-aldrich. BPO was purchased from Sigma Aldrich as the 98 wt% Luperox. Enriched ^{13}C-labeled MMA and MeOH were purchased form Cambridge Isotope Labs. All chemicals were used out of the bottle with no prior purification.

Synthesis of Dual Cure materials

For a typical synthesis of PECAN(50)-PMMA(45)-PMAA(5) at a 20 g scale, we would mix 10 g of pre-mixed PECAN monomers (3.9 g Erisys-60, 1.6 g of Erisys-21, and 4.5 g of MHHPA) with 9.5 g of MMA, 0.5 g of MAA, 0.10 g of BPO, and 0.10 g of 24EMI (it is easiest to first dissolve BPO and 24EMI in a methacrylate before mixing with PECAN) in a 100 mL schlenk flask equipped with a stir bar. The Schlenk flask was then connected to a firestone valve. Vacuum was applied to the until bubble formation (boiling) was observed. The mixture was given about a minute to stir with boiling at which point the Schlenk was refilled with N_2 gas. This was repeated 3-4 times before injecting 0.10 mL of DMAn to start the reaction. The reaction mixture was then poured or distributed into a suitable container (Teflon mold, reactor, or vial) to cure. Some measures were taken to prevent exposure to O_2, such as keeping the reaction in a sealed vial filled with either N_2 or Ar in the headspace. These reactions were typically given 2-4 h at RT for the methacrylate polymerization, and then put in an oven set to 80 ^oC for 4 h. In some cases, especially when mechanical testing was the objective, a 160 ^oC post-cure was used for 30 min.

The "ziplock-bag" method was used with the Teflon molds for DMA and tensile coupons. This followed the above procedure; the degassed monomer mixture was poured into the molds and slipped into a ziplock bag. The bag contained a metal frame to keep the roof of the bag from drooping into the reaction. Nitrogen was purged into and out of the bag for about a minute to remove O_2 and the bag was sealed.

Calorimetry experiments were also prepared by the above method but at a very large scale (200 g) and was poured from the Schlenk flask into a reactor. This reactor was covered by a blanket of Ar (bloxygen), a temperature probe was dipped into the liquid and the headspace was covered.

Synthesis of ^{13}C-labeled MMA, PECAN(50)-PMMA(50), and PECAN(50)-PMMA(45)-PMAA(5): MMA labelled at 2 different carbons were acquired for our NMR-morphology studies. The MMA ^{13}C-labelled at the carbonyl carbon was purchased and used as received, while MMA ^{13}C-labeled at the methyl ester was synthesized in-house. For this synthesis, 1 g of ^{13}C-labeled methanol was dissolved in 100 mL of methylene chloride (first dried over 3 Å molecular sieves for several days) along with 5 g of triethylamine (dried over CaH_2 overnight) in a 200 mL Schlenk flask. This mixture was sealed with a rubber septum and subjected to several vacuum/N_2 purges to ensure an air-free environment, then cooled to zero ^oC using an ice bath. Methacroyl chloride (5 g, 1.53 molar equiv) was sparged from a sure-seal bottle into an oven-dried syringe and then injected into the reaction solution through the rubber septum, over the course of 5 min. The ice bath was removed and the reaction allowed to warm to RT. After 24 h, the reaction was quenched

with aqueous saturated $NaHCO_3$. Using a separatory funnel, the organic layer was isolated and washed 3 additional times with aqueous $NaHCO_3$ and once with brine. The organic layer was then concentrated down to about 10 mL, and then columned using a combiflash NEXTGEN 300+ with pure DCM as the mobile phase and a RediSep Gold 80 g HP silica column as the stationary phase. The chromatogram (Figure S1, left) revealed a major product (MMA) and 1 minor product (presumably methacrylic anhydride, MAAn) with good separation between the two. Appropriate fractions were combined, and DCM was carefully removed by rotary evaporation to finally furnish 1.3 g of ^{13}C-labeled PMMA (~42 % yield) confirmed by H NMR (Figure S1, right).

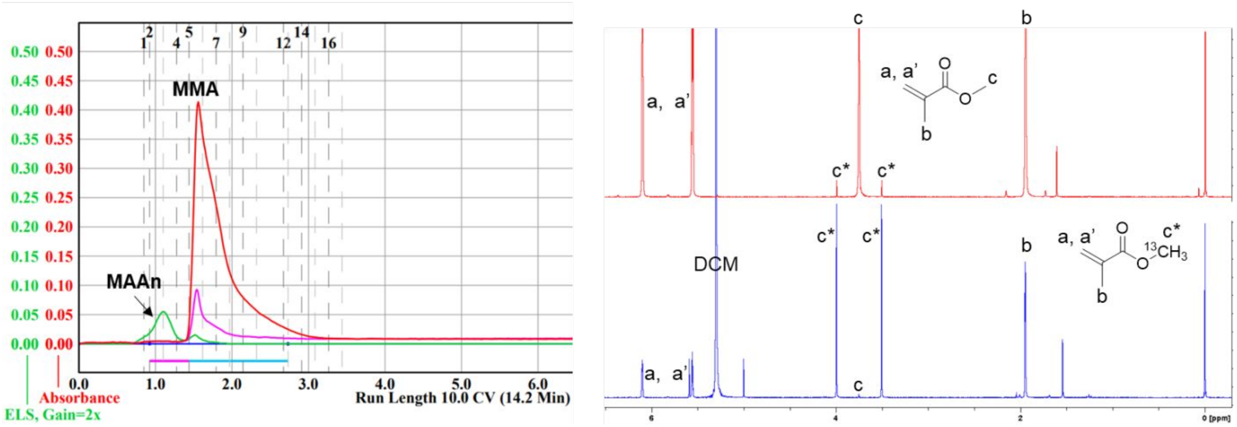

Figure S1: **Purification and Identification of 13C-labeled MMA.** (**Left**) CombiFlash chromatogram of ^{13}C-labeled MMA product purification and (**right**) H NMR of 13C-Labeled MMA (bottom spectrum) overlayed with a natural abundance sample of MMA (top spectrum).

In preparation for the synthesis of ^{13}C-labeled PECAN(50)-PMMA(50) and PECAN(50)-PMMA(45)-PMAA(5), a 1/1/1 v/v/v mixture of [$^{13}C_{carbonyl}$/$^{13}C_{methyl\ ester}$/Natural abundance] MMA was prepared. This mixture was used to synthesize 1 g of each PECAN(50)-PMMA(50) and PECAN(50)-PMMA(45)-PMAA(5) according to the standard method.

Analytical Methods

NMR Spectroscopy: H NMR spectra were obtained using a Bruker 300 MHz, typically using $CDCl_3$ and referenced to $C\mathit{H}Cl_3$ peak at 7.26 ppm.

Solid-state NMR Spectroscopy: All solid-state NMR experiments were performed using a 200 MHz (4.7 Tesla) Bruker AvanceIII HD NMR spectrometer equipped with a 4 mm HX double-resonance MAS probe. All samples were finely ground and packed into 4 mm zirconium rotors and capped with Kel-F drive tips. Samples were spun at the magic angle at 10 kHz. To be consistent with ssNMR best practices, ^{13}C chemical shifts were referenced externally to TMS at 0 ppm by setting the downfield Adamantane signal to 38.48 ppm.[1] The bearing gas was pre-cooled and the temperature at the probe was maintained at 10 °C. For Cross Polarization on samples at natural abundance, the following conditions were used: an initial 2.6 μs proton hard pulse followed by a 2 ms CP contact time where the ^{13}C channel was set to 62.5 kHz field strength and the ^{1}H channel was matched to the +1 spinning sideband of the Hartman-Hahn profile using a 10% ramped spin-lock pulse. 4096 scan averages were collected using a 3 second recycle delay, and 80 kHz ^{1}H high power decoupling (Bruker sequence swftppm13) during a 20 ms acquisition time. Proton relaxation in the laboratory frame (^{1}H T_1) values were measured indirectly through proximal carbons using the CP inversion recovery method (cphirt1 Bruker pulse sequence), 256 scan averages for each of the 8 points time points, while proton relaxation in the rotating frame (^{1}H $T_{1\rho}$) were collected using a 62.5 kHz variable length ^{1}H spin-locking pulse prior to routine CP and acquisition (cpht1rho Bruker sequence) using 256 scan averages for each 8 spin-lock times. Relaxation parameters for each ^{13}C spectral region were extracted by fitting the signal area vs. time to known functions.

For selective one-dimensional (1D) ^{13}C-^{13}C spin-diffusion measurements on ^{13}C-enriched thermosets we used a similar approach as previously described.[2,3] Namely, we used the MultiCP method[4] to quantitatively excite all ^{13}C sites prior to a storage pulse, selective inversion and spin diffusion. Two datasets were collected: selective 1D MultiCP-DARR with spin-diffusion mixing times ranging from 0.001 ms to 7000 ms, and a non-selective counterpart at identical mixing times. MultiCP conditions were as follows: 3.5 us ^{1}H and ^{13}C hard pulses, a total of 5 CP steps (62.5 kHz ^{13}C and 14% ramp on the ^{1}H channel) of 1100 μs each, separated by a 0.8 second t_z repolarization delay. A hard storage pulse was applied after MultiCP to store ^{13}C magnetization along +z. For the selective MultiCP-DARR dataset, selective inversion of the O-CH_3 signal at 52 ppm was achieved using a 10 ms Gaussian cascade Q3 inversion pulse,[5] while this step is skipped in the non-selective dataset. In both cases, the ^{13}C-^{13}C spin-diffusion mixing period utilized DARR mixing where a continuous wave pulse on the ^{1}H channel was applied at the first rotary resonance condition ($\omega_1 = \omega_r$).[6] Comparing the selective and non-selective datasets at each mixing time provides the fraction of ^{13}C sink carbon sties that reside within “spin diffusion range” of the selected source.[3] Data were processed using MestreNova version 14 and analysed using custom python code.

Dynamic Scanning Calorimetry: DSC was performed using a DSC TI-Q2000 with T0 hermitic aluminum pans. Isotermal experiments followed a procedure such as ramp temperature to 80 °C from RT at 20 °C/min, then hold at 80 °C for 100 minutes.

Dynamic Mechanical Analysis: DMA was performed using a TA Q800 in single cantilever mode. DMA bars were synthesized using the ziplock bag method (vide supra). Testing dimensions were

similar to 30 mm × 13.5 mm × 3.0 mm. Temperature sweeps followed a regiment between 30-180 °C at 5 °C/min with 30 μm displacement, 1 Hz. Creep tests were performed using the same coupons, again in single cantilever mode. The creep regiment involved soaking the material at a specified temperature for 10 min, then applying a 5 MPa stress for 10 minutes and measuring the strain response. This was repeated on the same coupon, for several temperatures (25-105 °C at 5 °C intervals).

Microscopy: SEM imaging was performed on an FEI Quanta 400 FED microscope (FEI, Hillsborough, OR, USA) under low vacuum (0.40 - 0.65 Torr) operating in conjunction with the gaseous solid-state detector (GAD) collecting secondary electrons. Samples were prepared by cryo-fracturing rectangular films following 2 minutes of soaking to obtain unperturbed morphology in the cross-section. Dried fractured samples were then adhered to an aluminum stub using double-sided carbon tape and sputter-coated with 10 nm iridium. Imaging was performed at beam accelerating voltages of 30 kV.

Brief Discussion on ssNMR Theory and Methodology, and Results.

Solid-state NMR (ssNMR) proton spin relaxation data was used to probe PECAN/PMMA polymer miscibility on the ~2 - 50 nm length-scale. Proton spin-lattice (^{1}H T_1) and rotating frame (^{1}H $T_{1\rho}$) relaxation rates in the solid state are sensitive to nano-scale separation of domains; an averaging of ^{1}H relaxation rates due to efficient ^{1}H-^{1}H spin-diffusion between domains is indicative of polymer miscibility over the length scales defined by the experiment.[7] The length-scales over which efficient spin-diffusion manifests are well known:

$$< x > = \sqrt{2\varepsilon D t}$$

Where <x> is the root mean square distance (RMSD), ε is a prefactor governed by the sample morphology, defined as 3 for cubes or spheres in a matrix, D is the spin-diffusion coefficient, and t is the time-scale during which spin-diffusion occurs. Spin-diffusion coefficients for rigid organic polymers can vary system to system, but are generally <= 0.8 nm^2/ms for larger domains,[7] or ~0.2 - 0.3 nm^2/ms for localized domains via $T_{1\rho}$ measurements.[8] ^{1}H T_1 and $T_{1\rho}$ values for PECAN and PMMA are on the order of 0.5-1 second and ~10 milliseconds, respectively (Table S1-S2). Therefore, ^{1}H T_1 relaxation is sensitive to spin-diffusion induced averaging over domains in the range of 30-60 nm, while ^{1}H $T_{1\rho}$ rates are sensitive to spin-diffusion effects on the ~2-3 nm scale.

Therefore to investigate polymer miscibility on the tens of nanometer and 2-3 nm length-scales, ^{1}H T_1 and $T_{1\rho}$ were measured for four samples: PMMA(100), PECAN(100), PECAN(50)-PMMA(50), which is macro-phase-separated based on SEM, and PECAN(50)-PMMA(45)-PMAA(5), which is hypothesized to be more homogeneous and is clearly absent of any observable macro-phase separation by SEM analysis. Consistent with this hypothesis, we observed ^{1}H T_1 (Table S1) and $T_{1\rho}$ (Table S2) averaging for the PECAN(50)-PMMA(45)-PMAA(5) sample but insignificant averaging for the phase-separated PECAN(50)-PMMA(50).

Average ^{1}H T_1 values for neat PMMA and PECAN were 0.49 and 0.90 seconds, respectively. The unique T_1 values of the isolated polymers indicates ^{1}H T_1 can be used to investigate polymer miscibility on the 30-60 nm length scales, since ^{1}H spin-diffusion will result in averaging of relaxation rates if polymers are sufficiently mixed. ^{1}H T_1 for PECAN(50)PMMA(50) shows evidence of separate domains, since ^{1}H T_1 values are substantially different between the polymers. PMMA has shorter average ^{1}H T_1 values due to the increased relative abundance of methyl

relaxation sinks. When comparing ^{1}H relaxation rates associated with resolved PMMA and PECAN signals in the phase-separated mixture to those from isolated polymers, approximately 9% averaging is observed on the 30-60nm length scale as defined by the spin-diffusion range. Most likely the small degree of T_1(H) averaging occurs at the interface of the separated domains, which must be much larger than 30-60 nm. Large domain sizes are consistent with TEM results. When the trojan horse is included we observe a near-complete averaging of ^{1}H T_1 values, with ^{1}H T_1 assignable to PMMA and PECAN structures converging to 0.62 and 0.65 s, respectively. Table S1 summarizes these results. PMMA and PECAN are therefore near-completely mixed on the 30-60 nm length scale.

Regarding rotating frame relaxation, polymerized PECAN(50)PMMA(50) $T_{1\rho}$ values detected via resolved ^{13}C sites also suggest phase-separation; we observe longer $T_{1\rho}$ for protons associated with PMMA compared to PECAN. ^{1}H T_{1p} for PECAN(50)PMMA(45)PMAA(5) shows evidence of polymer mixing on the 2-3 nanometer scale, since ^{1}H T_{1p} values are fairly well averaged. Note that for the 50:45:5 "homogeneous" sample PMMA signals have shorter T_{1p} and PECAN has longer T_{1p} compared to "heterogeneous" sample (T_{1p} values move towards the average). The degree of mixing is not confidently decerned due to the increased diversity of $T_{1\rho}$ rates. However, the averaged $T_{1\rho}$ value for all protons within the "homogeneous" sample is 12 ms, while signals associated with PMMA and PECAN show averaged $T_{1\rho}$ values of 11.9 and 13.0 ms, respectively (Table S2). Taken as a proxy, these $T_{1\rho}$ rates might indicate near-complete polymer mixing on the 2-3 nm scale.

Table S1: ^{1}H Spin-Lattice Relaxation Times in the Laboratory Frame (^{1}H T_1). Values are obtained via their associated carbons after ^{1}H-^{13}C cross-polarization using the CP inversion recovery method. The asterisks indicate spectral regions that significantly overlap and therefore are not included in the average calculations.

		^{1}H T1 (s)			
**^{13}C Shift**	**Polymer**	**PurePECAN**	**PurePMMA**	**50:50**	**50:45:5**
178	PMMA	-	0.48	0.56	0.64
173	PECAN	0.91	-	0.88	0.67
71	PECAN	0.91	-	0.87	0.65
56	PMMA	-	0.48	0.52	0.58
52	PMMA	-	0.49	0.55	0.64
45	PMMA*	-	0.48	0.62	0.64
43	PECAN*	0.89	-	0.79	0.69
33	PECAN	0.89	-	0.89	0.62
29	PECAN	0.91	-	0.85	0.62
23	PECAN	0.91	-	0.77	0.67
17	PMMA	-	0.49	0.55	0.63
178, 56, 52, 17	Average PMMA	-	0.49	0.55	0.62
173, 71, 33, 29, 23	Average PECAN	0.90	-	0.85	0.65

Table S2: ^{1}H Spin-Lattice Relaxation Times in the Rotating Frame (^{1}H $T_{1\rho}$). The asterisks indicate spectral regions that significantly overlap and therefore are not included in the average calculations.

		^{1}H T1ρ (ms)			
**^{13}C Shift**	**Polymer**	**PurePECAN**	**PurePMMA**	**50:50**	**50:45:5**
178	PMMA	-	11.1	11.1	10.0
173	PECAN	7.0	-	10.1	19.4
71	PECAN	9.6	-	8.4	11.6
56	PMMA	-	14.3	14.1	12.1
52	PMMA	-	12.9	16.0	12.2
45	PMMA*	-	13.9	18.7	10.1
43	PECAN*	10.9	-	25.9	9.3
33	PECAN	10.5	-	10.5	10.9
29	PECAN	9.9	-	12.6	10.9
23	PECAN	8.4	-	10.6	11.9
17	PMMA	-	9.9	26.6	13.3
178, 56, 52, 17	Average PMMA	-	12.1	17.0	11.9
173, 71, 33, 29, 23	Average PECAN	9.1	-	10.4	13.0

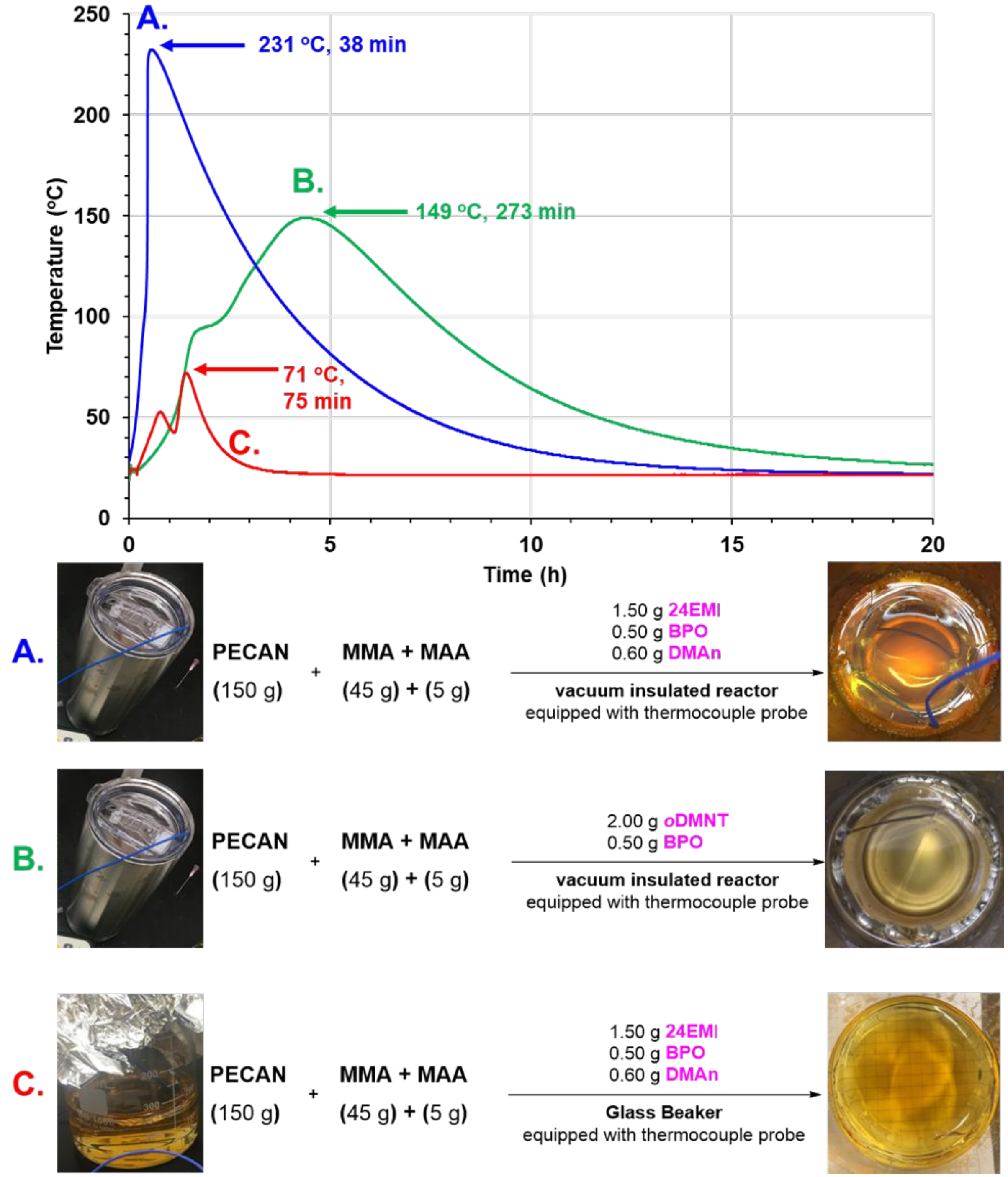

Figure S2: **Large Scale Field Tests of PECAN/MMA DC Reactions by Chemical Heating**. Thermograms of three different dual cure conditions, all using the same formulation PECAN(75)-PMMA(22.5)-PMAA(2.5), with (A, blue) using the highly active initiation system BPO/DMAn and 24EMI (here DMAn initiates free radical polymerization while 24EMI initiates epoxy/anhydride ring-opening) in a vacuum insulated reactor, (B, green) using sluggish initiation system oDMNT/BPO (here, oDMNT initiates both free radical polymerization and epoxy/anhydride ring-opening), and (C, red) highly active DMAn/BPO and 24EMI in a non-insulating glass beaker.

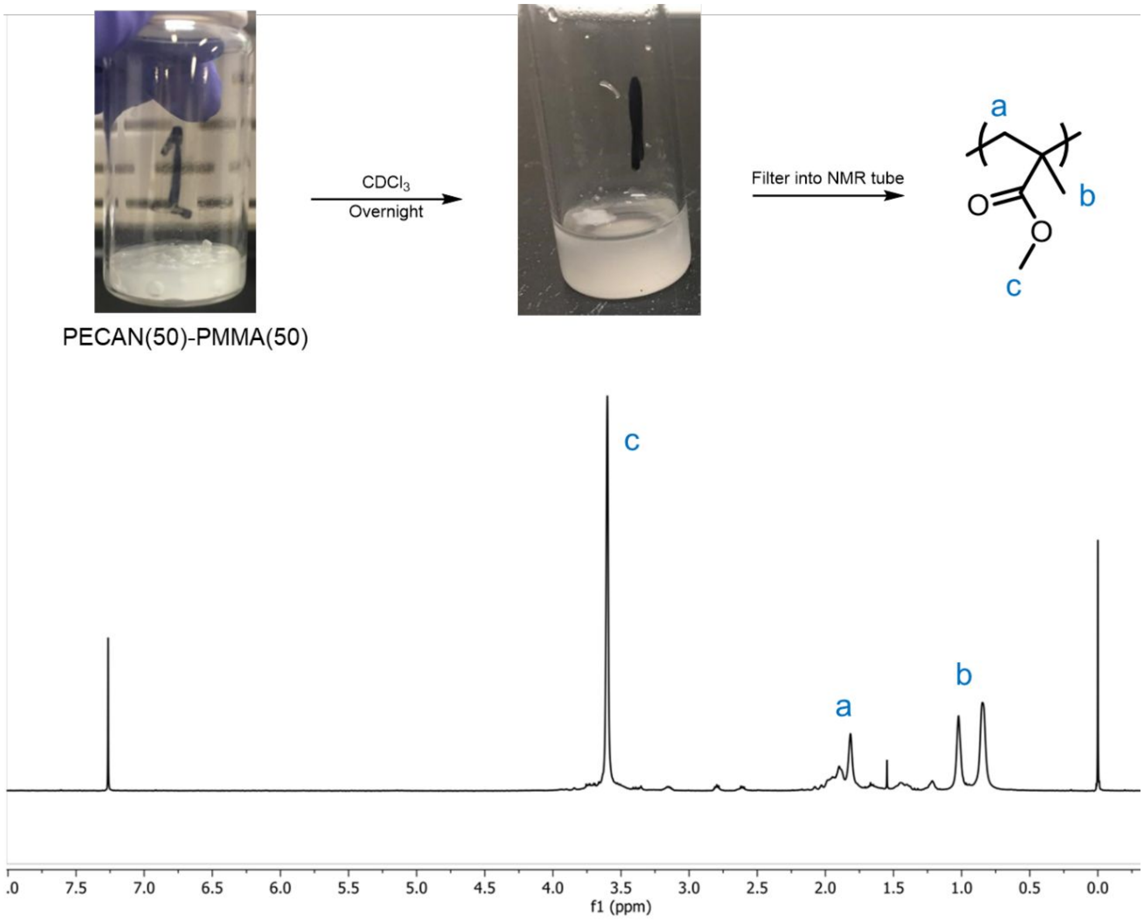

Figure S3: DC Material Extraction Experiment (No Trojan Horse). H NMR spectrum of PMMA extracted from PECAN(50)-PMMA(50) by simple overnight soak in $CDCl_3$, showing non-covalent linkage between PECAN and PMMA domains.

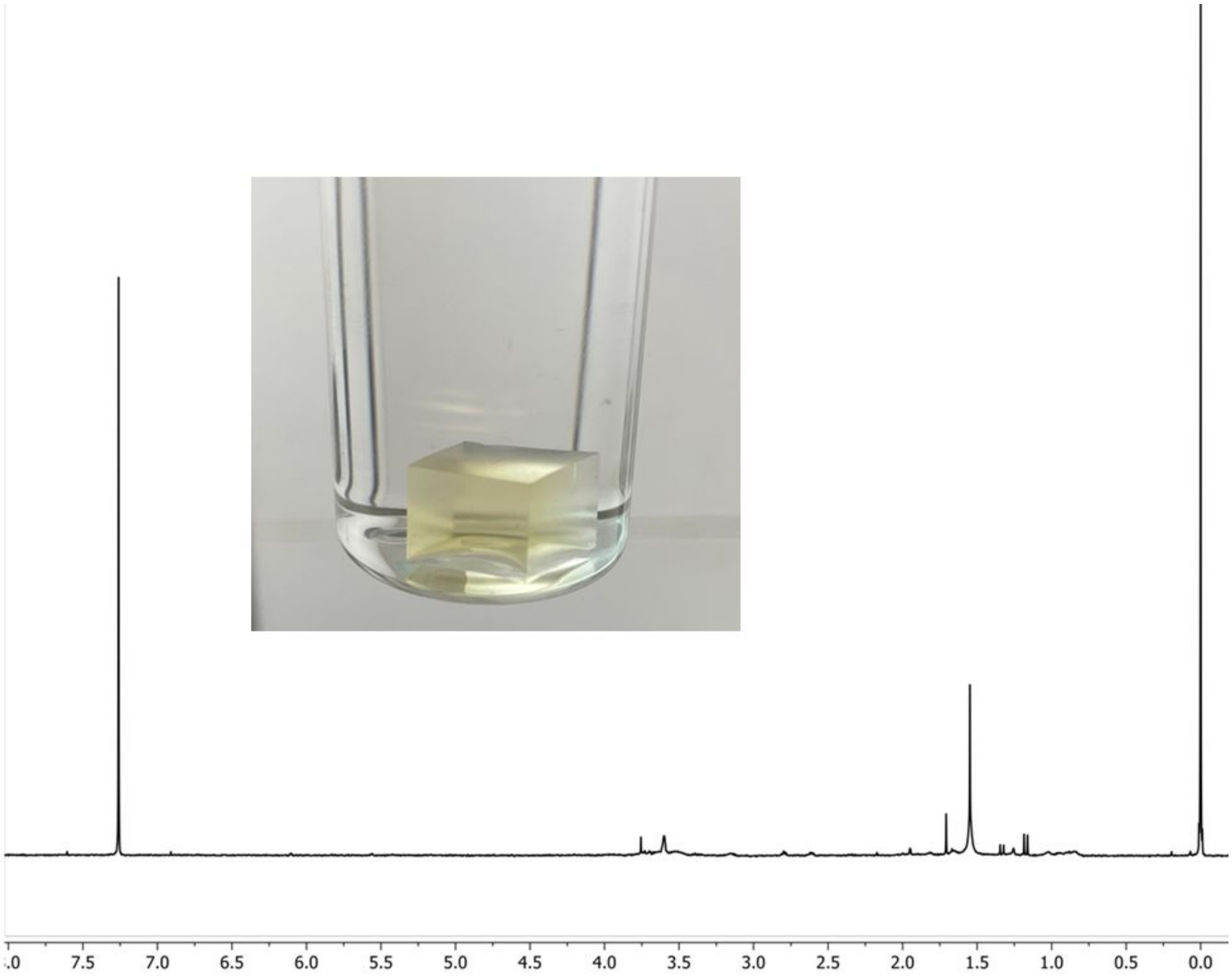

Figure S4: **DC Material Extraction Experiment (Trojan Horse).** Blank H NMR spectrum showing lack of products extracted from PECAN(50)-PMMA(45)-PMAA(5) by simple overnight soak in $CDCl_3$, implying covalent linkages binding PECAN to PMMA domains.

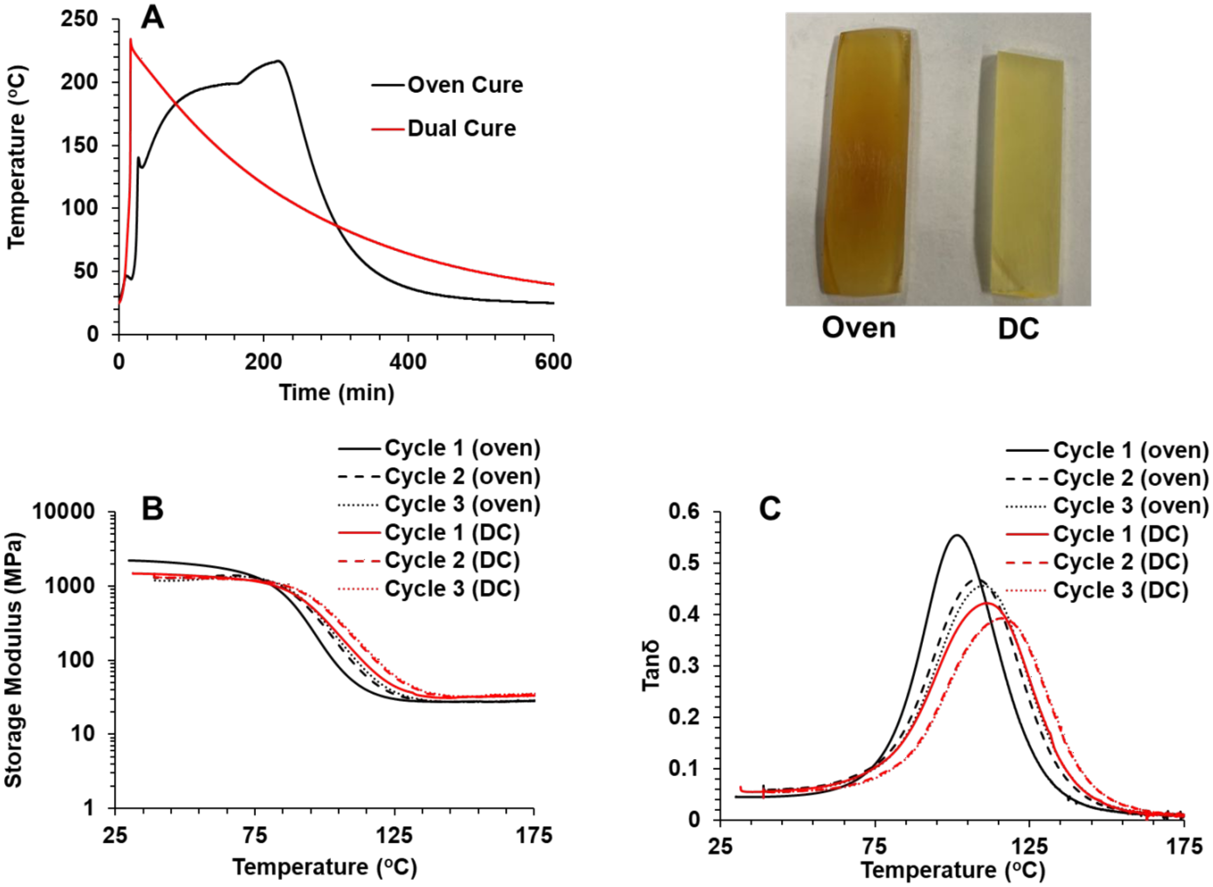

Figure S5. DMA plots of Oven Cure vs Dual Cure samples (see main text Figure 6) over multiple cycles. (A) Thermograms of the cure schedules for Oven Cure vs Dual Cure samples as well as a photograph of the tested coupons. (B) Storage modulus vs temperature plot of Oven Cure vs Dual Cure samples showing three cycles, meant to emphasize differences in material properties upon multiple heating cycles after neutralizing thermal history and further curing samples in-situ. (C) Tanδ vs temperature plot of Oven Cure vs Dual Cure samples showing three cycles, meant to emphasize differences in material properties upon multiple heating cycles after neutralizing thermal history and further curing samples in-situ.

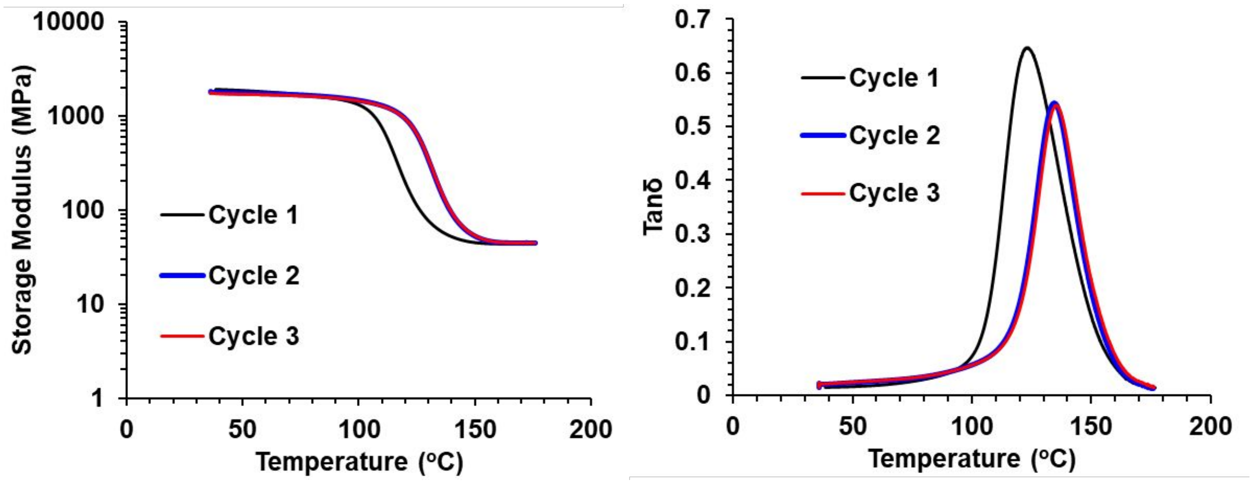

Figure S6. DMA plots for Run 1 showing all 3 cycles (see main text Table 1 and Figure 7). Emphasized here is that following cycle 1, cycles 2 and 3 are stable and unchanging. Thus we assume the cure is complete and the thermal history neutralized so that different samples can be compared on the basis of cycle 2 (Cycle 2 is what is shown in main text Figure 7).

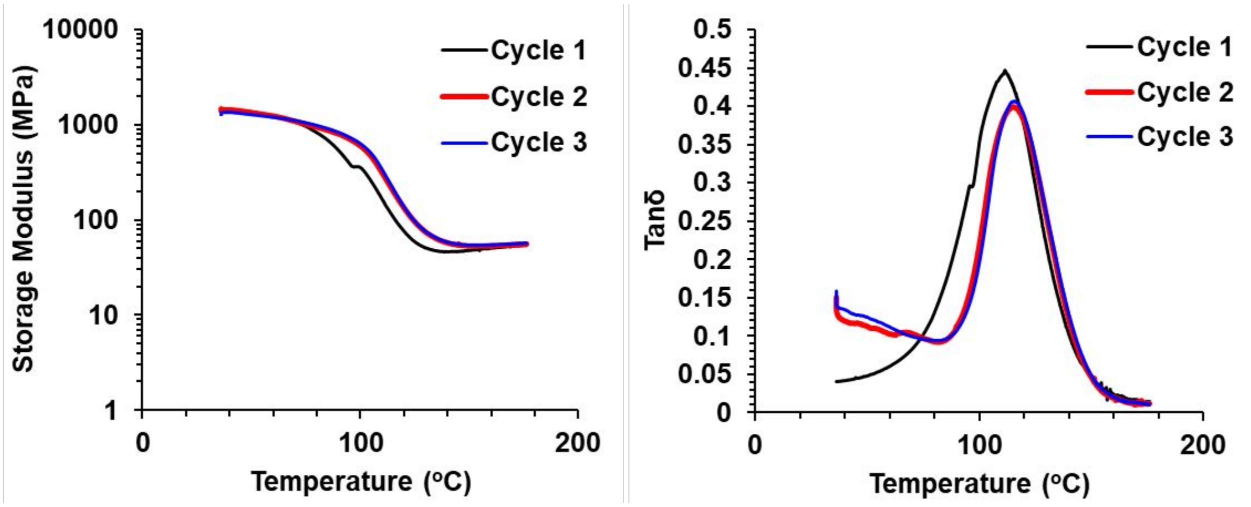

Figure S7. DMA plots for Run 6 showing all 3 cycles (see main text Table 1 and Figure 7). Emphasized here is that following cycle 1, the material is stable and the strange tanδ behavior is reproducible upon multiple cycles.

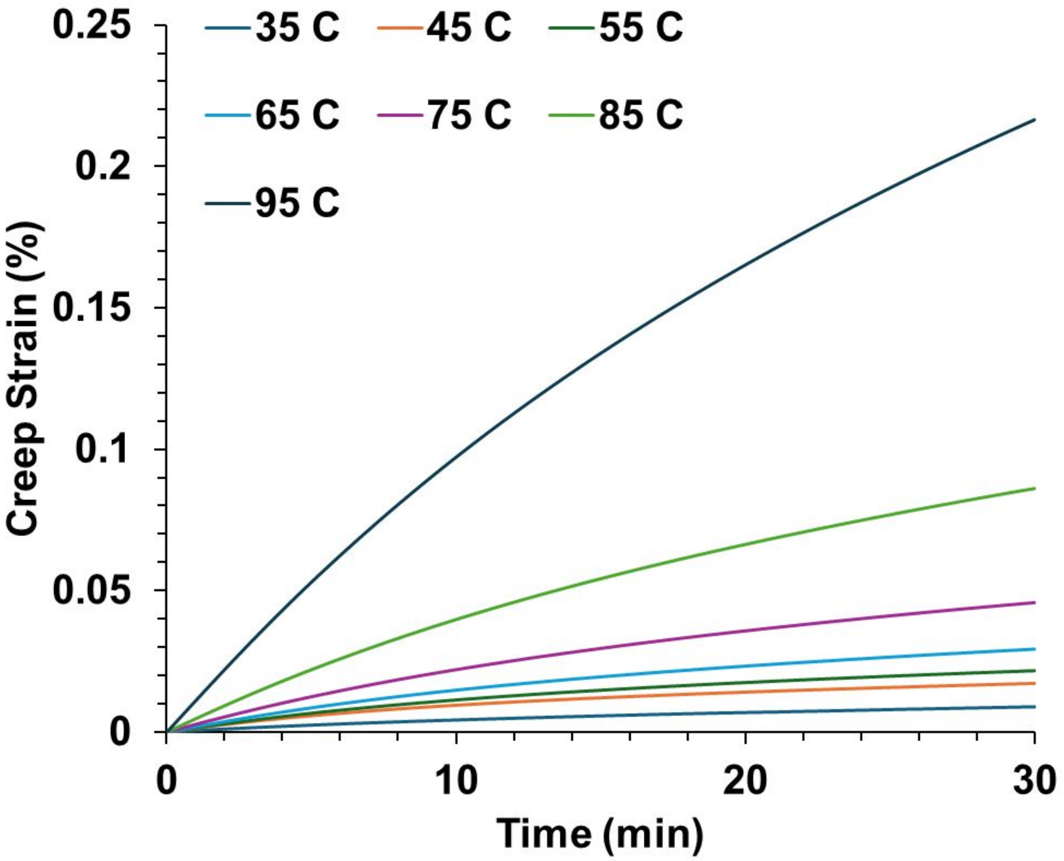

Figure S8. An example strain vs time plot corresponding to the creep tests performed on Run 1 (See Main text Table 1 and Figure 7). This plot was made for each run and was used to calculate the steady state strain rate (SSSR) for each time point by approximating the slope of each curve between 25-30 min.

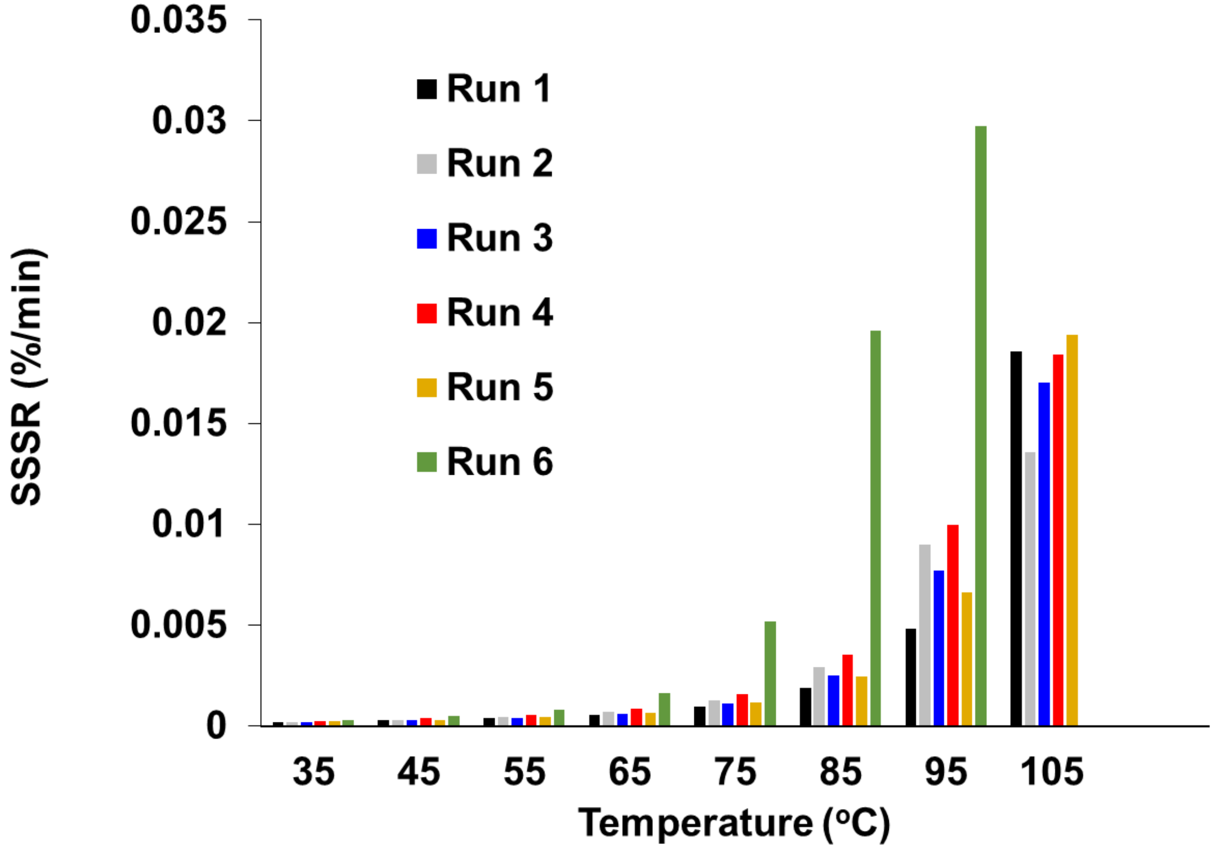

Figure S9. Creep tests for Runs 1-6 (see main text Table 1 and Figure 7)**.** Shown is the steady state strain rate (SSSR) of each material at different temperatures.

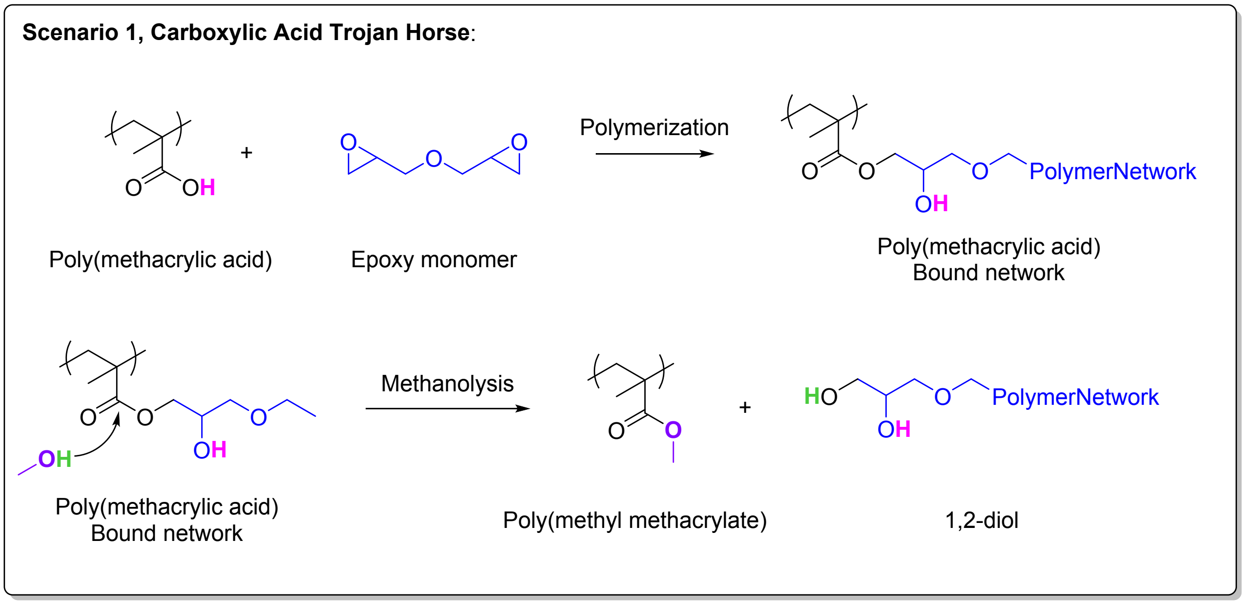

Figure S10. Infered methanolysis chemistry hypothesized to deconstruct DC materials employing carboxylic acid trojan horse. Products rendered include small molecule diols, small molecule methyl esters, and poly(methyl methacrylate).

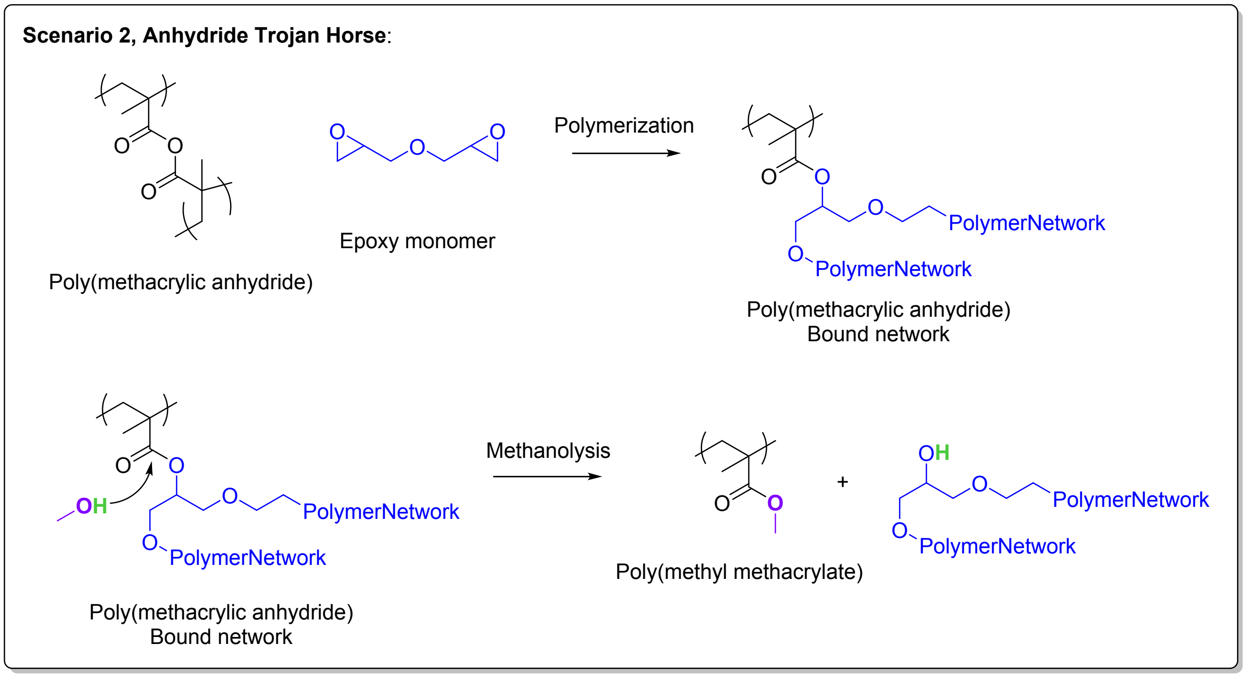

Figure S11. Infered methanolysis chemistry hypothesized to deconstruct DC materials employing anhydride trojan horse. Products rendered include small molecule diols, small molecule methyl esters, and poly(methyl methacrylate).

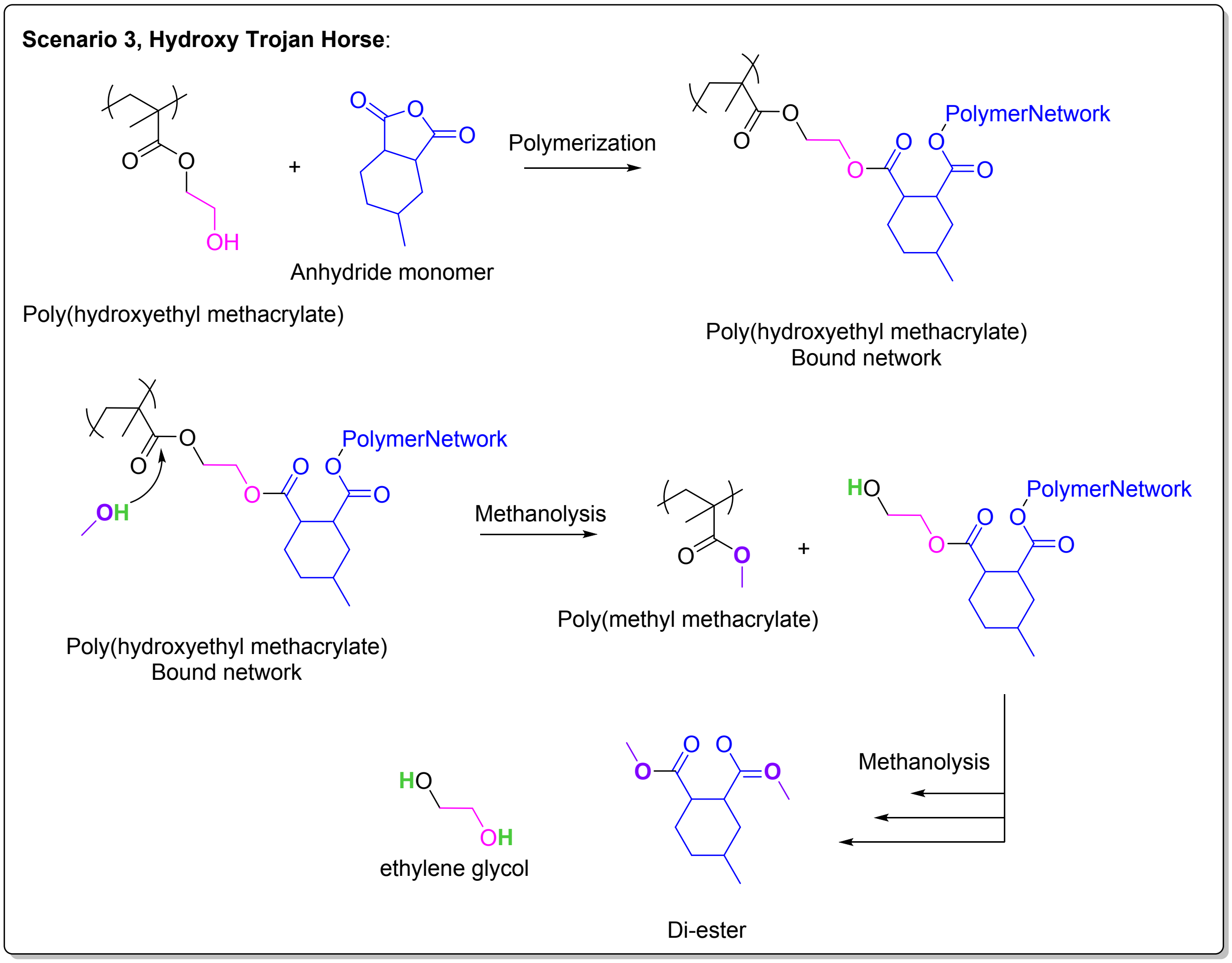

Figure S12. Infered methanolysis chemistry hypothesized to deconstruct DC materials employing hydroxy trojan horse. Products rendered include small molecule diols, small molecule methyl esters, and poly(methyl methacrylate).

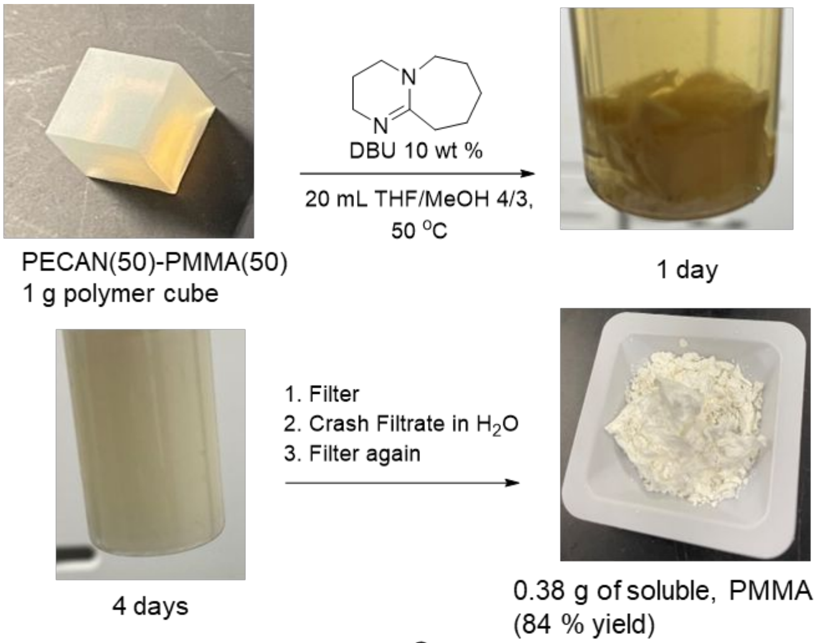

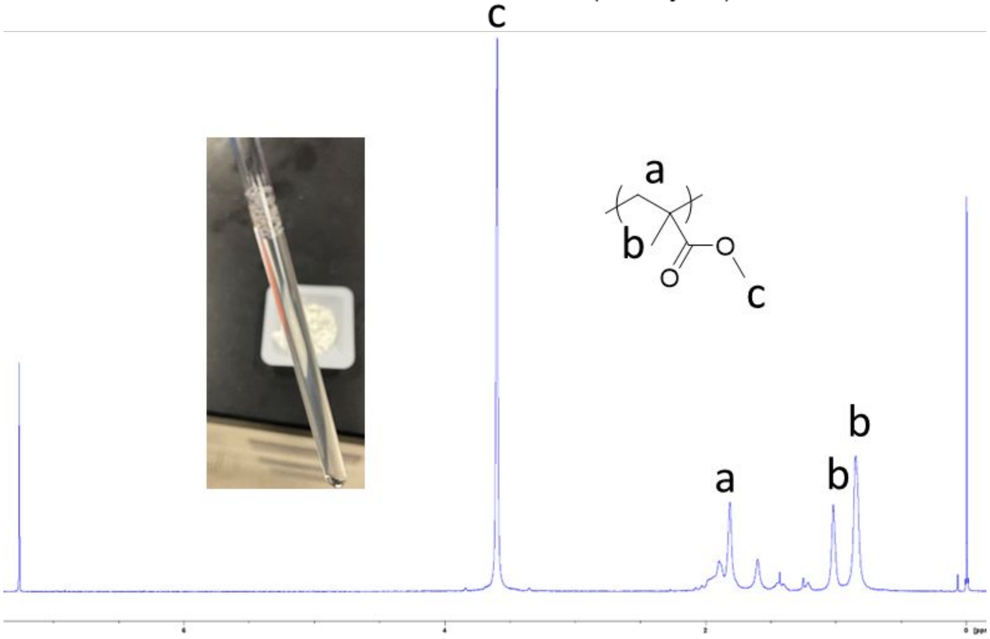

Figure S13. Deconstruction of PECAN(50)-PMMA(50) and H NMR of Recovered PMMA.

References

1 Morcombe, C. R. & Zilm, K. W. Chemical shift referencing in MAS solid state NMR. *Journal of Magnetic Resonance* **162**, 479-486 (2003). https://doi.org:https://doi.org/10.1016/S1090-7807(03)00082-X

2 Addison, B. *et al.* Selective One-Dimensional 13C–13C Spin-Diffusion Solid-State Nuclear Magnetic Resonance Methods to Probe Spatial Arrangements in Biopolymers Including Plant Cell Walls, Peptides, and Spider Silk. *The Journal of Physical Chemistry B* **124**, 9870-9883 (2020). https://doi.org:10.1021/acs.jpcb.0c07759

3 Addison, B. *Science Advances* **in press** (2024).

4 Johnson, R. L. & Schmidt-Rohr, K. Quantitative solid-state 13C NMR with signal enhancement by multiple cross polarization. *Journal of Magnetic Resonance* **239**, 44-49 (2014). https://doi.org:https://doi.org/10.1016/j.jmr.2013.11.009

5 Emsley, L. & Bodenhausen, G. Gaussian pulse cascades: New analytical functions for rectangular selective inversion and in-phase excitation in NMR. *Chemical Physics Letters* **165**, 469-476 (1990). https://doi.org:https://doi.org/10.1016/0009-2614(90)87025-M

6 Takegoshi, K., Nakamura, S. & Terao, T. 13C–1H dipolar-assisted rotational resonance in magic-angle spinning NMR. *Chemical Physics Letters* **344**, 631-637 (2001). https://doi.org:https://doi.org/10.1016/S0009-2614(01)00791-6

7 Clauss, J., Schmidt-Rohr, K. & Spiess, H. W. Determination of domain sizes in heterogeneous polymers by solid-state NMR. *Acta Polymerica* **44**, 1-17 (1993). https://doi.org:https://doi.org/10.1002/actp.1993.010440101

8 Chen, Q. & Schmidt-Rohr, K. Measurement of the local 1H spin-diffusion coefficient in polymers. *Solid State Nuclear Magnetic Resonance* **29**, 142-152 (2006). https://doi.org:https://doi.org/10.1016/j.ssnmr.2005.09.008
